# Liquid phase synthesis of aromatic poly(azomethine)s, their physicochemical properties, and measurement of *ex situ* electrical conductivity of pelletized powdered samples

**DOI:** 10.1080/15685551.2016.1231042

**Published:** 2016-09-23

**Authors:** Abdul Hafeez, Zareen Akhter, John F. Gallagher, Humaira M. Siddiqui

**Affiliations:** ^a^ Department of Chemistry, Quaid-i-Azam University, Islamabad, Pakistan; ^b^ School of Chemical Sciences, Dublin City University, Dublin, Ireland

**Keywords:** Polyazomethines, *bis*-aldehydes, monomers, single crystals, conductivity, poly Schiff bases

## Abstract

Aromatic *bis*-aldehydes have been used as building blocks in the synthesis of polyazomethines (a class of conjugated Schiff bases) and their physicochemical properties have been studied. Six dialdehydes have been synthesized, **3a-3f**, via etherification reaction between aromatic diols (**2a-2f**) and 4-fluorobenzaldehyde (**1**) (see Scheme 1), and then polymerized with 1,4-phenylenediamine (**4a**) and 4,4′-oxydianiline (**4b**) (see Scheme 2). The chemical structures of the *bis*-aldehydes were elucidated by FTIR, ^1^H NMR and ^13^C NMR spectroscopic studies, elemental analysis and single crystal whereas the polymers were studied by FTIR and NMR spectroscopy. Their physicochemical properties were examined by their inherent viscosity, organosolubility, differential scanning calorimetry, X-ray powder diffraction, thermogravimetric analysis, solvatochromism, and photoluminescence. We report the electrical conductivity of each polymer measured by the four probe method. The results indicate that the electrical conductivity of polymers lies in range 0.019–0.051 mScm^−1^ which is reasonably higher than any reported value.

## 1. Introduction

Conducting polymer based semiconductors have become a dominant platform for modern electronics.[1] The expensive and complicated vacuum processes used for the preparation of metal-oxide based semiconductors restricts their scope of application.[2] Therefore, cost effective, low temperature processable materials are required and conducting polymers are one of the potential candidates for the development of such materials.[3] Conducting polymers have a number of advantages over inorganic compounds such as outstanding coloration efficiency, multiple colors with the same material, fast switching ability, and fine-tuning of the band gap (and color) through chemical structure modification.[4] However, there are some inherent drawbacks in conducting polymers as well, such as low material recovery and stability to environments, lack of film uniformity over large surface, and irregular linkages within the polymer backbone chain.[5,6]

As one class of conducting polymers, polyazomethines (PAM) (with the azomethine linkage in the polymer backbone) have attracted considerable attention, for example, the azomethine linkage is a renowned mesogen in liquid crystalline polymers.[7,8] The –C(H)=N– bond of PAM is isoelectronic with the –CH=CH– group and has a similar planar molecular structure with the –CH=CH– group.[9] As PAM type systems are capable of being protonated and complexed with metals, by taking the advantage of these characteristics, PAM materials have potential applications in electronics, optoelectronics, and photonics.[10,11] The increased interest in this class of polymers is due to their high thermal stability, metal chelating ability, excellent mechanical strength, semi-conducting, and excellent optoelectronic properties.[12] Owing to these brilliant properties they are used to prepare composites, graphite materials, epoxy oligomer, photoresists, and block copolymers.[13,14] In terms of synthesis, it is expedient to synthesize PAM by polycondensation of the appropriate amine and carboxaldehyde. Moreover, their resulting purification is relatively easier and they can be purified with soxhlet extraction and/or reduced pressure drying. The synthetic methods and purifications of many other π-extended conjugation polymers [15] necessitate expensive monomer, stern conditions, and noble catalysts.[16] Therefore, in this article we report the syntheses and characterization of a series of PAM synthesized from aromatic bisaldehydes in order to provide superior properties of semi-electrical conduction and with very high thermal stabilities resulting from of the introduction of additional aromatic rings. Our group has already carried out studies on the design and synthesis of ferrocene based polyazomethine esters for multifunction applications.[17,18] The chemical structures of the monomers were characterized by FT-IR, NMR spectra, single crystal methods, and elemental analysis, while the polymers were analyzed by FT-IR, NMR spectra, and other physicochemical methods such as wide angle X-ray diffraction, UV–vis, differential scanning calorimetry (DSC), viscometry, thermogravimetric analyses (TGA), organosolubility, and solvatochromism. The synthetic approach of these polymers primarily focused on studying the effect on electrical conduction by changing the monomer and/or substituents on monomer, thereby allowing the possibility, to study their influence on the thermal, and photoluminescence (PL) properties. Macroscopic measurements of *ex situ* electrical conductance of bulk polymer powders is done either by two-probe or four-probe methods.[19] The optimal way to measure the conductance of a macroscopic polymer samples is four probes which exclude the resistance of the probes and the probe–polymer interfaces.[20,21]

## 2. Experimental

### 2.1. Materials

All chemicals used in the synthesis of bis-aldehydes and PAM were purchased from commercial suppliers and were of high purity. Hydroquinone (m.p. = 172–175 °C), resorcinol (m.p. = 110 °C), pyrocatechol (m.p. = 100–103 °C), 4-fluorobenzaldehyde (98%), 44′-dihydroxybiphenyl (97%, m.p. = 284–285 °C), bisphenol A (m.p. = 158–159 °C), (111,333-hexafluoro)–bisphenol propane (m.p. = 160–163 °C) were obtained from Sigma Aldrich. The chemicals K_2_CO_3_ anhydrous (Fluka), 14-phenylenediamine (m.p. = 138–143 °C, Sigma Aldrich), 44′-diaminodiphenyl ether (97%, m.p. = 188–192 °C, Sigma Aldrich), *p*-toluene sulfonic acid monohydrate (PTSA) were purchased from Fluka (Switzerland). The solvents *n*-hexane, dimethylsulfoxide, and diethyl ether were procured from Merck (Germany) whereas chloroform, ethylacetate, and toluene were purchased from Panreac (Spain). The ethanol, methanol, and *N*,*N*-dimethylformamide were obtained from Deijing (Korea) while hydrochloric acid, and sulfuric acid were procured from Riedel-de-Haen (Germany). The solvents used in this work were dried where necessary with standard procedures.[22]

### 2.2. Measurements

Melting points of *bis*-aldehydes were determined on a Mel-Temp (Mitamura Riken Rogyo, Inc. Tokyo, Japan) apparatus using open capillary tubes and are uncorrected. Fourier transform infrared (FTIR) spectra of samples were recorded with Perkin Elmer 1600 series FTIR spectrophotometer. Nuclear magnetic resonance measurements were undertaken using a Bruker Avance 300 digital NMR in different solvents depending upon their solubility as mentioned with NMR data of each sample given below in the text and with tetramethylsilane as internal standard. Elemental analyses were performed on a Vaio-EL instrument. The mass spectra of the monomers were recorded on Agilent 5973 inert GC-MS System with column (J & W Scientific, 30 m × 0.25 mm × 0.5 μm) at a temperature 120–280 °C with heating rate 10 °C/min. The UV–vis spectra of azomethine polymers were measured on Schimadzu-1700 UV using CHCl_3_, DMF, and sulfuric acid. TGA were run on Perkin Elmer TGA instrument v.7 at a heating rate of 10 and 20 °C/min in nitrogen or air to a maximum 650–950 °C. PL spectra of the PAM were measured on Perkin Elmer LS 55 luminescence instrument using single glass cell. DSC studies were performed employing Mettler Toledo DSC 823E instrument at heating rate of 10 °C/min a nitrogen flow rate of 50 mL/min. The viscosities of PAM were determined at room temperature employing U-tube Ubbelhode viscometer with 20 mL solutions. Wide-angle X-ray diffraction measurements of powdered polymers were done at 298 K on Philips 3040/60 X’Pert Pro diffractometer having Cu anode with-Kα radiation source parameters were constrained. The electrical conductivity (*σ*) of PAM was measured with Keithley source meter 2400. The single crystal X-ray diffraction of two monomers **3a** and **3e** at 294 K were determined using methods previously published by our research group in DCU (**SI**).[23,24] Refinements were initially performed using SHELXL14/7.[25] Diagrams were generated using the PLATON and Mercury programs (Figures 4 and 5).[26,27] CSD analyses were performed using version 5.36 of the CSD and WebCSD.[28,29]

### 2.3. Syntheses

**Scheme 1.**
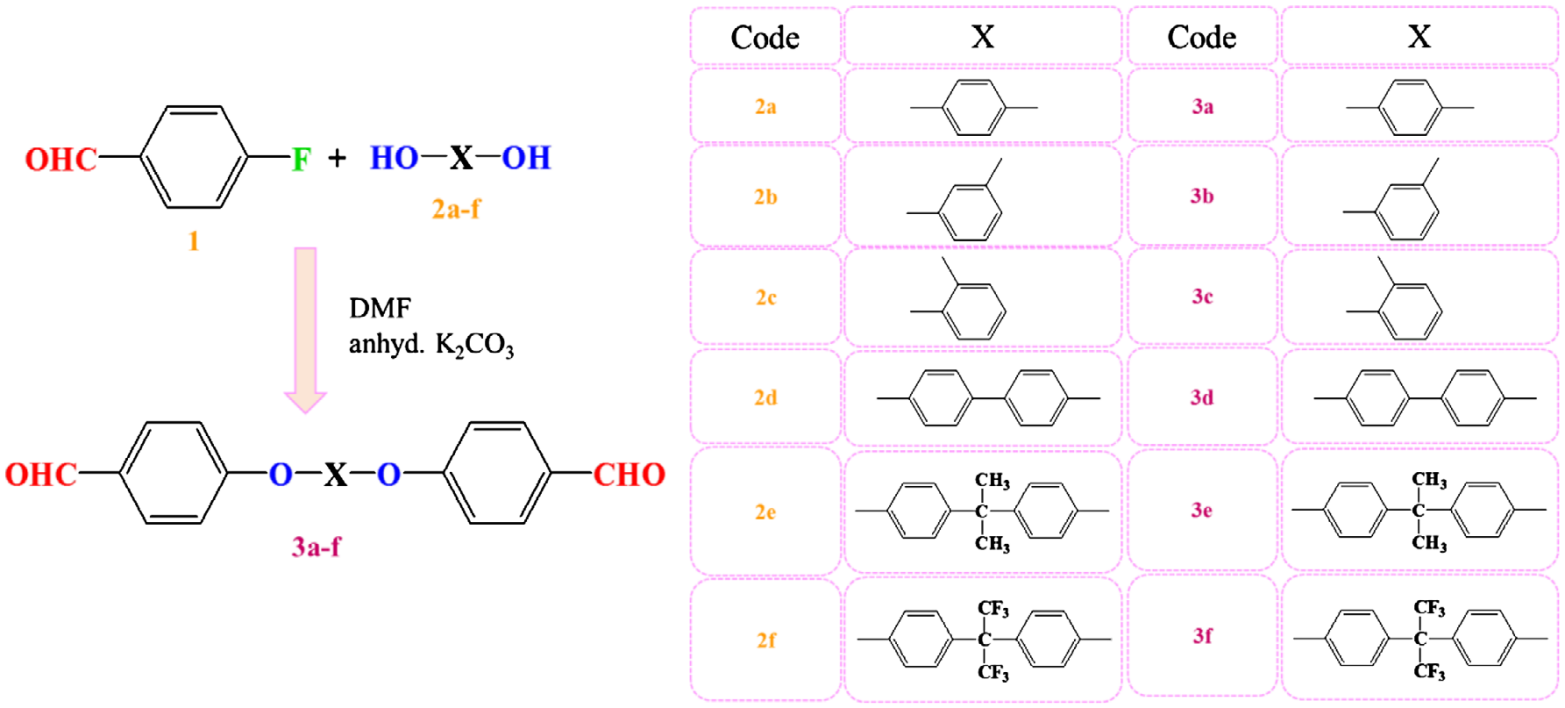
Aromatic bis-aldehydes synthesized by etherification reaction*.*

**Scheme 2.**
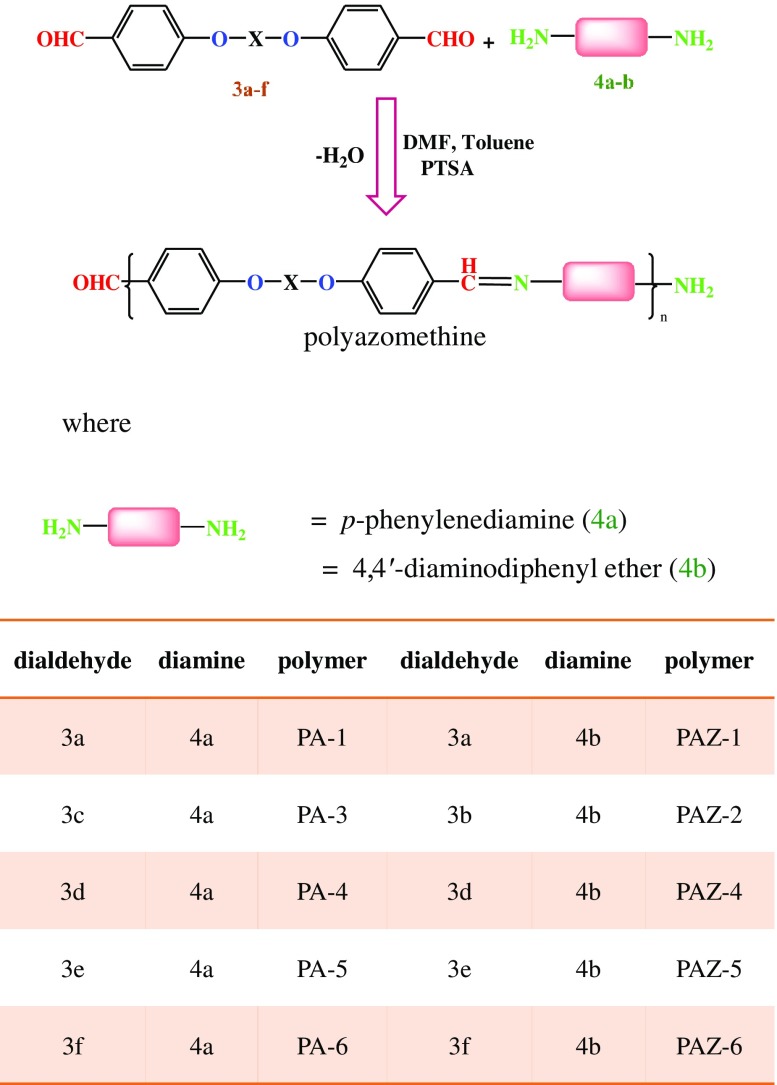
Synthesis of polyazomethines.

The chemical structures and synthetic schemes for monomers and polymers are described in Schemes 1 and 2, respectively. The spectral data and salient crystal structural information of two monomers can be found in the Electronic Supplementary Information.

#### 2.3.1. Syntheses and characterization of monomers

The Scheme 1 depicts how six aromatic *bis*-aldehyde monomers (**3a-f**) were synthesized by simple one step etherification between 4- fluorobenzaldehyde (**1**) and several different dihydroxybenzenes (**2a-f**).

#### 2.3.2. Synthesis of 4,4′-[1,4-phenylene-bis-oxy]bis-benzenecarboxaldehyde (3a)

The synthesis of monomer **3a** is used to illustrate the general procedure. A prebaked 100 mL two necked round bottom flask was equipped with magnetic stirrer, 0.025 mol (2.75 g) of hydroquinone, 0.06 mol (8.0 g) anhydrous K_2_CO_3_ and 40 mL freshly dried DMF. The mixture was heated for 1 h at 80–90 °C under an inert atmosphere, then cooled to *ca*. 40 °C followed by the addition of 5.4 mL 4-fluorobenzaldehyde (0.05 mol). The stirring was continued for 45 min, followed by heating the mixture for 50 h at 154 °C. In order to check the reaction progress several TLC runs were performed in ethyl acetate (EtOAc) and *n*-hexane in different ratios. Upon completion of the reaction the contents of reaction flask were poured into 350 mL ice cold water. After neutralizing the base with 10% HCl solution the precipitates were collected. Subsequently, the product mixture was filtered, washed with H_2_O, oven-dried and recrystallized with ethanol/ethyl acetate/chloroform solvent mixture followed by drying to afford the di-ether based aromatic bisaldehyde **3a**.

Yield, 91%, m.p. 153–155 °C. FT-IR (*ʋ* = cm^−1^): 3029 cm^−1^ (aromatic C–H str.); 2727 cm^−1^ (aldehydic C–H str.); 1692 cm^−1^ (C=O str.); 1599, 1489 cm^−1^ (aromatic C=C); 1225, 1187 cm^−1^ (C–O–C ether): ^1^H NMR (300 MHz, CDCl_3_) δ 9.94 (s, 2H), 7.88 (d, *J* = 8.7 Hz, 4H), 7.15 (s, 4H), 7.10 (d, *J* = 8.6 Hz, 4H). ^13^C NMR (75 MHz, CDCl_3_) δ 190.76 (s), 163.09 (s), 151.86 (s), 132.04 (s), 131.44 (s), 122.04 (s), 117.50 (s). Elem. Anal. Calc.: C_20_H_14_O_4_: C, 75.46; H, 4.43; O, 20.10. Found: C, 76.06; H, 3.94%. EIMS (*m/z*): 318 [M]^+^, 317 [M–H]^+^, 289 [M–CHO]^+^, 261 [M–C_3_H_5_O]^+^, 196 [C_13_H_8_O_2_]^+^, 168 [C_12_H_8_O]^+^, 77 [C_6_H_5_]^+^, 51 [C_4_H_3_]^+^.

##### X-ray crystallographic data of 3a

The crystals of **3a** were grown by slow evaporation of an acetone/CHCl_3_ solution. C_20_H_14_O_4_, *M* = 318.31, space group P2_1_/c, *a* = 9.7949(4), *b* = 10.6069(5), *c* = 15.1758(6) Å, *α* = 90°, *β* = 97.783(4)°, *γ* = 90°, *V* = 1562.14(12) Å^3^, *Z* = 4, *Z*′ = 1, R-factors (%) 4.74 and 0.1292.

#### 2.3.3. Synthesis of 4,4′-[1,3-phenylene-bis-oxy]bis-benzenecarboxyaldehyde (3b)

The same protocol was used for **3b** as was used for **3a**. Yield, 82%, m.p. 108–110 °C. FT-IR (*ʋ* = cm^−1^): 3063 cm^−1^ (aromatic C–H str.); 2829, 2733 cm^−1^ (aldehydic C–H str.); 1687 cm^−1^ (carbonyl str.); 1577, 1497 cm^−1^ (aromatic C=C); 1210, 1148 cm^−1^ (C–O–C ether): ^1^H NMR (300 MHz, CDCl_3_) δ 9.94 (s, 2H), 8.06–7.73 (m, 4H), 7.60–6.44 (m, 8H). ^13^C NMR (75 MHz, CDCl_3_) δ 190.85 (s), 162.38 (s), 156.74 (s), 132.13 (s), 131.89 (d, *J* = 26.4 Hz), 131.26 (s), 119.39 (s), 118.06 (s), 116.28 (s), 112.19 (s). Elem. Anal. Calc.: C_20_H_14_O_4_: C, 75.46; H, 4.43; O, 20.10. Found: C, 76.06; H, 3.94%. EIMS (*m/z*): 318 [M]^+^, 317 [M–H]^+^, 290 [M–CO]^+^, 289 [M–CHO]^+^, 262 [M–C_3_H_4_O]^+^, 196 [C_13_H_8_O_2_]^+^, 168 [C_12_H_8_O]^+^, 141 [C_10_H_6_O]^+^, 115 [C_8_H_4_O]^+^, 77 [C_6_H_5_]^+^, 51 [C_4_H_3_]^+^.

#### 2.3.4. Synthesis of 4,4′-[1,2-phenylene-bis-oxy]bis-benzenecarboxaldehyde (3c)

The procedure used for **3a** was also used for **3c**. Yield, 77%. FT-IR (*ʋ* = cm^−1^): 3057 cm^−1^ (aromatic C–H str.); 2825, 2733 cm^−1^ (aldehydic C–H str.); 1687 cm^−1^ (carbonyl str.); 1583, 1488 cm^−1^ (aromatic C=C); 1259, 1213 cm^−1^ (C–O–C ether): ^1^H NMR (300 MHz, CDCl_3_) δ 9.86 (s, 2H), 7.76 (d, *J* = 8.5 Hz, 4H), 7.27 (dt, *J* = 9.6, 5.8 Hz, 4H), 6.88 (d, *J* = 8.5 Hz, 4H). ^13^C NMR (75 MHz, CDCl_3_) δ 190.76 (s), 162.39 (s), 146.19 (s), 131.82 (s), 131.38 (s), 126.72 (s), 123.26 (s), 116.66 (s). Elem. Anal. Calc.: C_20_H_14_O_4_: C, 75.46; H, 4.43; O, 20.10. Found: C, 76.06; H, 3.94%. EIMS (*m/z*): 318 [M]^+^, 317 [M-H]^+^, 290 [M-CO]^+^, 289 [M-CHO]^+^, 108 [C_6_H_4_O_2_]^+^, 92 [C_6_H_4_O]^+^, 77 [C_6_H_5_]^+^, 51 [C_4_H_3_]^+^.

#### 2.3.5. Synthesis of 4,4′-di[(4-formylphenyl)oxy]biphenyl (3d)

A similar procedure was used for **3d** as was used for **3a** using 4,4′-dihydroxybiphenyl instead of hydroquinone. Yield, 84%, m.p. 160 °C. FT-IR (*ʋ* = cm^−1^): 3062 cm^−1^ (aromatic C–H str.); 2812, 2712 cm^−1^ (aldehydic C–H str.); 1688 cm^−1^ (carbonyl str.); 1592, 1487 cm^−1^ (aromatic C=C); 1250, 1207 cm^−1^ (C–O–C ether): ^1^H NMR (300 MHz, DMSO) δ 9.94 (s, 2H), 7.96 (d, *J* = 8.6 Hz, 4H), 7.79 (d, *J* = 8.6 Hz, 4H), 7.23 (dd, *J* = 17.8, 8.6 Hz, 8H). ^13^C NMR (75 MHz, DMSO) δ 192.05 (s), 154.79 (s), 136.46 (s), 132.54 (s), 131.86 (s), 129.03 (s), 121.10 (s), 118.21 (s). Elem. Anal. Calc.: C_26_H_18_O_4_: C, 79.17; H, 4.60; O, 16.23. Found: C, 76.93; H, 4.24%. EIMS (*m/z*): No peak was observed, probably the sample decomposed.

#### 2.3.6. Synthesis of 2,2-di [4-(4′-formylphenyloxy)phenyl] propane (3e)

The protocol discussed for **3a** applies to **3e** by replacing hydroquinone with bisphenol A. Yield, 79%, m.p. 112 °C. FT-IR (*ʋ* = cm^−1^): 3065 cm^−1^ (aromatic C–H str.); 2819, 2731 cm^−1^ (aldehydic C–H str.); 1686 cm^−1^ (carbonyl str.); 1587, 1494 cm^−1^ (aromatic C=C); 1235, 1208 cm^−1^ (C–O–C ether): ^1^H NMR (300 MHz, CDCl_3_) δ 9.93 (s, 2H), 7.86 (d, *J* = 8.6 Hz, 4H), 7.31 (d, *J* = 8.6 Hz, 4H), 7.05 (dd, *J* = 17.9, 8.7 Hz, 8H), 1.75 (s, 6H). ^13^C NMR (75 MHz, CDCl_3_) δ 190.83 (s), 163.27 (s), 152.95 (s), 147.12 (s), 131.97 (s), 131.19 (s), 128.46 (s), 119.90 (s), 117.54 (s), 42.45 (s), 31.02 (s). Elem. Anal. Calc.: C_29_H_24_O_4_: C, 79.80; H, 5.54; O, 14.66. Found: C, 76.49; H, 4.24%. EIMS (*m/z*): 317 [C_20_H_13_O_4_]^+^, 289 [C_19_H_13_O_3_]^+^, 119 [C_9_H_11_]^+^, 77 [C_6_H_5_]^+^, 51 [C_4_H_3_]^+^.

##### X-ray crystallographic data of 3e

The crystals of **3e** were grown by slow evaporation of a DMF solution. C_29_H_24_O_4_, *M* = 436.48, space group P-1, *a* = 7.5560(9), *b* = 11.762(2), *c* = 13.8277(18) Å, *α* = 95.137(12)°, *β* = 103.256(10)°, *γ* = 102.373(12)°, *V* = 11,155.9(3) Å^3^, *Z* = 2, *Z*′ = 1, R-factors (%) 5.48, 0.1540.

#### 2.3.7. Synthesis of 2,2-di [4-(4′-formylphenyloxy)phenyl]-1,1,1,3,3,3-hexafluoropropane (3f)

The **3f** was synthesized by the method used for **3a**. Yield, 73%, m.p. 90–94 °C. FT-IR (*ʋ* = cm^−1^): 3090 cm^−1^ (aromatic C–H str.); 2850, 2750 cm^−1^ (aldehydic C–H str.); 1686 cm^−1^ (carbonyl str.); 1588, 1494 cm^−1^ (aromatic C=C); 1232, 1205 cm^−1^ (C–O–C ether): ^1^H NMR (300 MHz, CDCl_3_) δ 9.96 (s, 2H), 7.91 (d, *J* = 8.6 Hz, 4H), 7.46 (d, *J* = 8.7 Hz, 4H), 7.13 (dd, *J* = 18.2, 8.8 Hz, 8H). ^13^C NMR (75 MHz, CDCl_3_) δ 190.73 (s), 161.80 (s), 156.14 (s), 132.04 (s), 129.14 (s), 119.27 (s), 118.61 (s). Elem. Anal. Calc.: C_29_H_18_F_6_O_4_: C, 63.98; H, 3.33; F, 20.94; O, 11.75. Found: C, 63.56; H, 3.32%. EIMS (*m/z*): 544 [M]^+^, 516 [M–CO]^+^, 318 [C_20_H_14_O_4_]^+^, 226 [C_9_H_4_F_6_]^+^, 168 [C_12_H_8_O], 77 [C_6_H_5_]^+^, 51 [C_4_H_3_]^+^.

### 2.4. Syntheses and characterization of polymers

#### 2.4.1. Syntheses of polymers

The synthesis of polymer **PA-1** was used as an example to illustrate the general protocol for the synthesis of polyazomethines. In a 100 mL two neck round bottom flask 0.1 mmol of **3a**, 20 mg of PTSA, a magnetic stirrer and 15 mL of DMF/toluene mixture in 2:1 ratio was taken. Stirred the solution until **3a** completely dissolved. Then dropwise added 0.1 mmol **4a** solution in DMF with dropping funnel under nitrogen atmosphere. When finished, the solution was gently heated for 6 h to distill for azeotropic removal of water using dean-stark trapper. After cooling to ambient temperature solution was poured into ice cold water and allowed standing for some time before filtration. Then washed with MeOH and dried in oven at 40 °C for 6 h before doing characterization. The rest of the polymers were synthesized using same method and are depicted in Scheme 2.

## 3. Results and discussions

### 3.1. Syntheses of monomers

**Figure 1.**
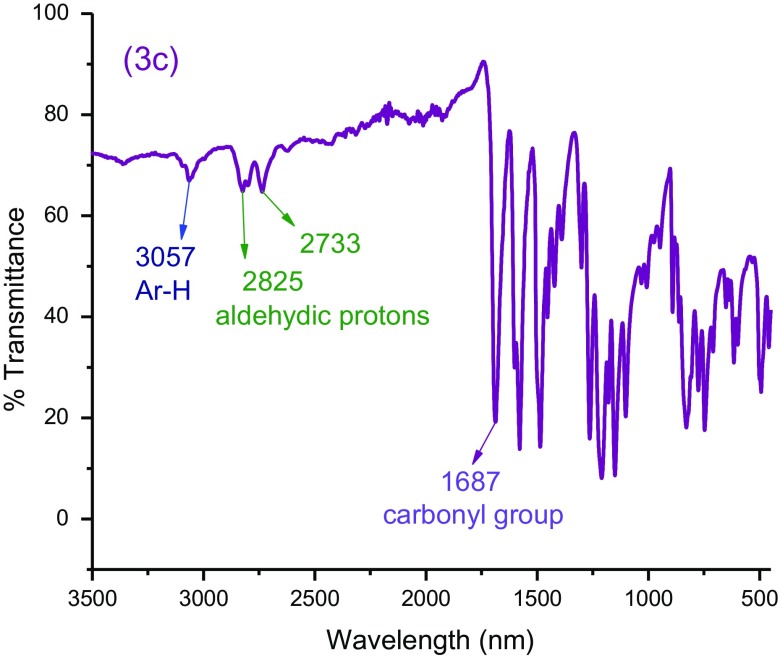
FT-IR spectrum of monomer 3c*.*

The spectral data and crystallographic information data (3a and 3e) of bis-aldehydes were found to be consistent with their molecular structures. Their chemical structures were identified with FT–IR, ^1^H–NMR, and ^13^C–NMR spectra. The FT-IR spectrum of monomer **3c** has been shown in Figure 1 which showed the distinct band at 1687 cm^−1^ that can be assigned to carbonyl group (C=O) of aldehyde whereas the bands at 3057 cm^−1^, and 2825, 2733 cm^−1^ are attributed to aromatic C–H stretching and aldehydic protons, respectively. The other significant bands at 1583, 1488 cm^−1^ are assignable to aromatic C=Cs, hence corroborating the proposed structures. Similarly the FT-IR spectra of all of the *bis*-aldehydes were in accordance with their proposed structures.

**Figure 2.**
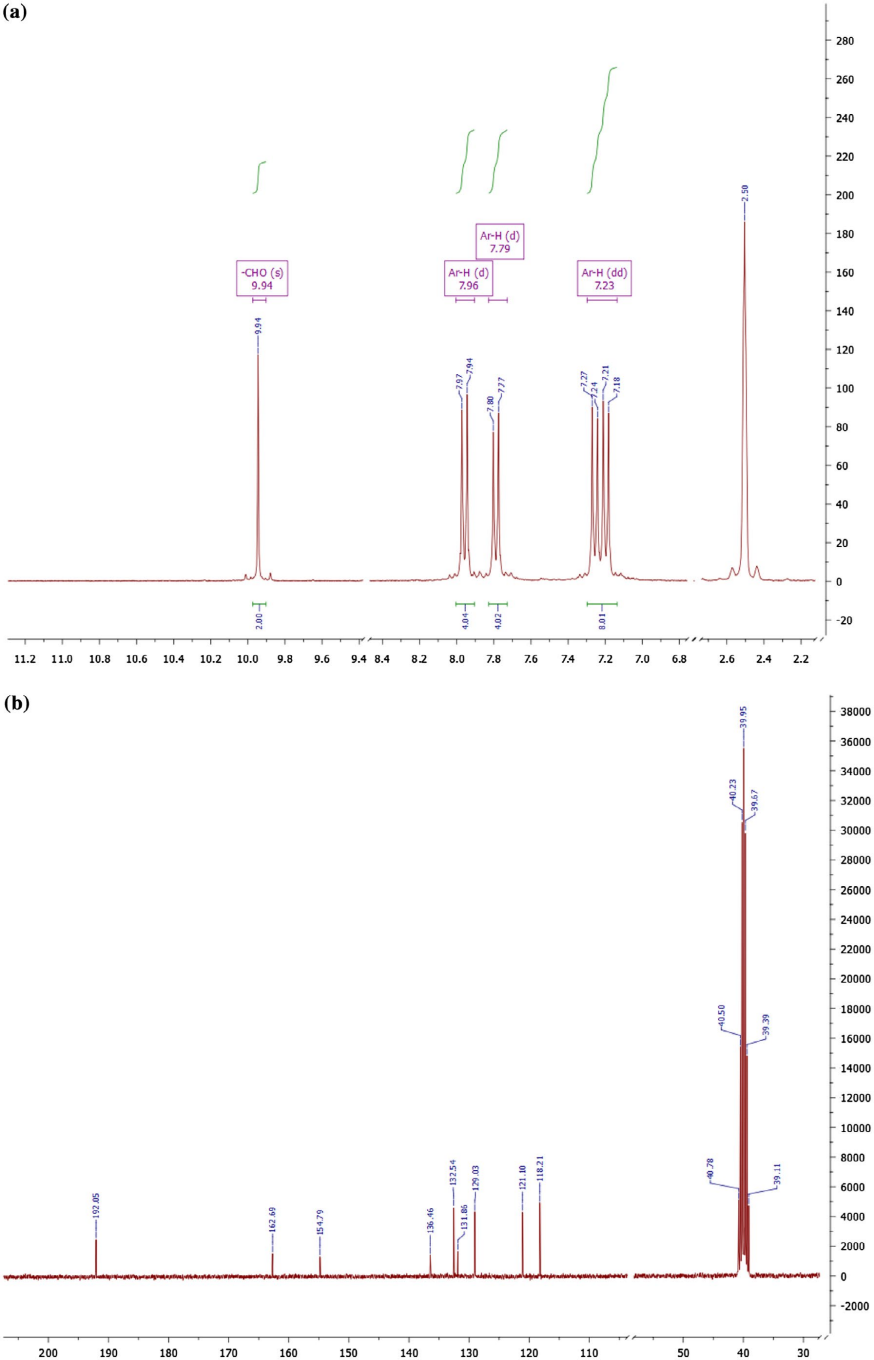
(a) Proton and (b) carbon NMR of monomer 3d in DMSO-d_6_ *.*

The Figure 2 shows the nuclear magnetic resonance spectra of monomer **3d** measured in DMSO-d_6_. In proton NMR (Figure 2(a)) a singlet at δ 9.94 ppm integrating two protons confirms the aldehyde group and doublets at δ 7.96 and 7.79 ppm integrating 4H each with coupling constant *J* = 8.6 Hz confirms the symmetrically substituted aromatic rings. The *dd* peaks appearing at δ 7.23 ppm are also associated to aromatic protons while the peak at δ 2.50 ppm is due to deuterated solvent DMSO-d_6_. In carbon NMR (Figure 2(b)) the single peak at δ 192.05 ppm is due to aldehydic carbon and the peaks from 154.79 to 118.21 ppm are all due to aromatic carbon atoms. The quintet at 39.95 ppm is assigned to DMSO solvent.

**Figure 3.**
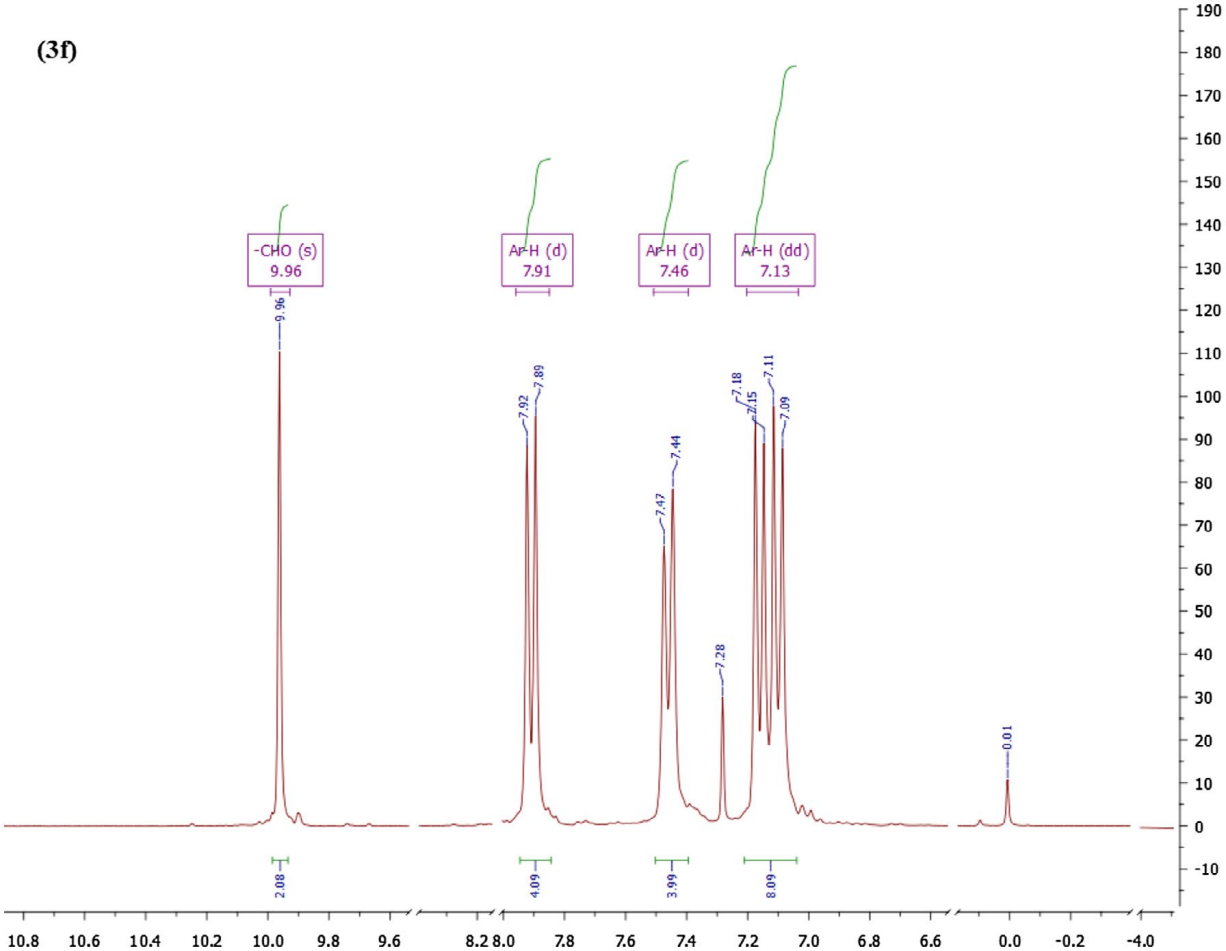
Proton NMR of monomer 3f in CDCl_3_ *.*

Similarly, the proton NMR spectrum of monomer **3f** measured in CDCl_3_ is shown in Figure 3 and the peaks are assignable as: singlet at δ 9.96 ppm is due to aldehydic protons whereas picturesque doublets at δ 7.91 (*J* = 8.6 Hz), and 7.46 (*J* = 8.7 Hz) integrating 4H each clearly confirm the symmetrically substituted aromatic rings. The peak at 7.28 ppm is because of chloroform whilst *dd* appearing at δ 7.13 ppm owing to 8H confirms para-linked aromatic rings. Likewise nuclear magnetic resonance spectra of the rest of bis-aldehydes were corroborating their structures.

### 3.2. Single crystal X-ray crystallographic analysis

Molecules of **3a** are non-planar by necessity as directly influenced by the two flexible –C_6_H_4_–O–C_6_H_4_– linkages. The three aromatic rings are at angles of 62.70(7)° and 71.84(6)° to one another whereas the terminal inter-planar angles are almost co-planar at an angle of 9.14(8)°. Views of the molecule **3a** in Figure 1. FT-IR spectra of monomer 3c.

**Figure 4.**
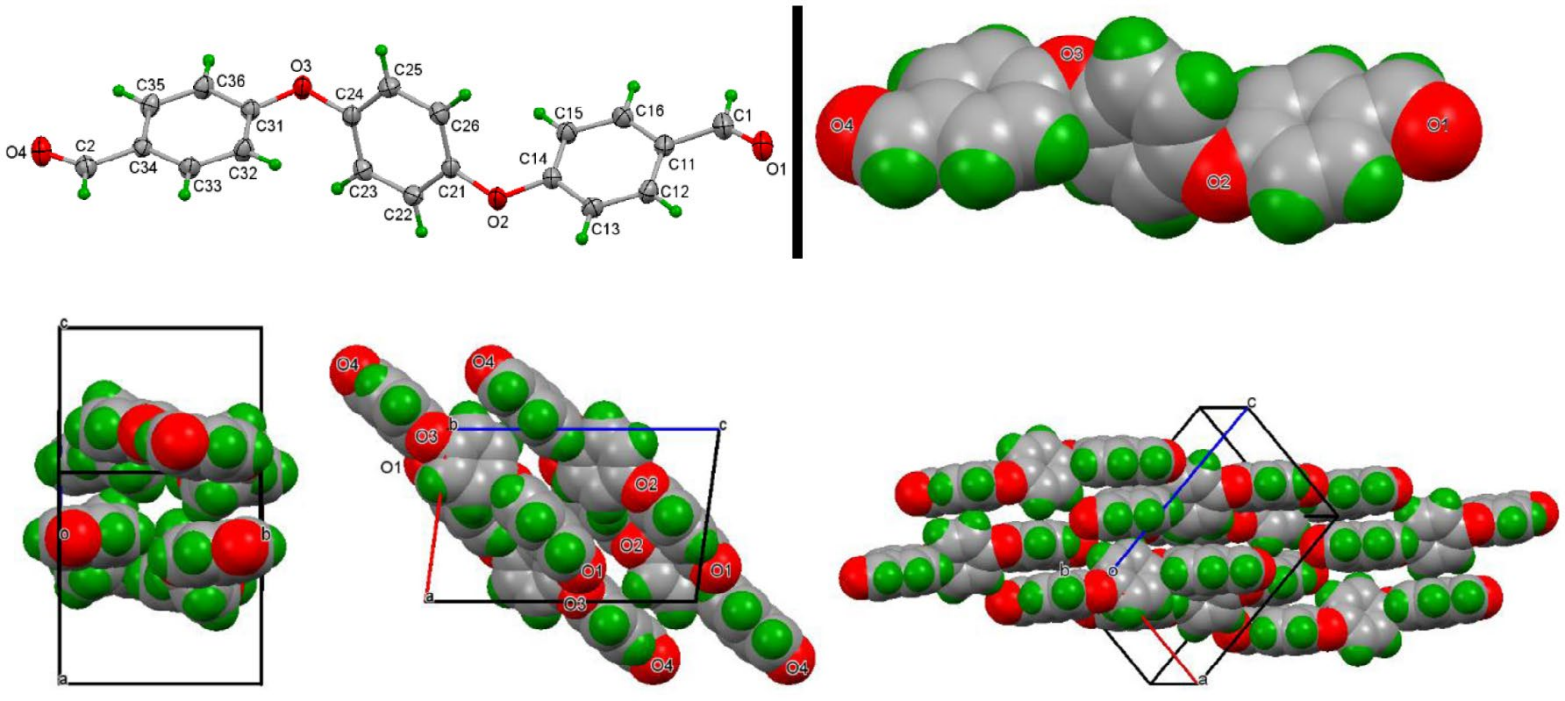
ORTEP view of **3a**; similar orientation with atoms as van der Waals spheres; three views of the unit cell highlighting the crystal structure defining intermolecular C–H … O and C–H … *π* interactions and an extended array of molecules*.*

Figure 4 show the molecular and crystal structure. Aggregation is by a short C26–H26 … O4^***i***^ interaction (symmetry code ***i* **= −1 + *x*, 3/2 − *y*, −1/2 + *z*) and a compact C–H … π interaction involving C22 with H22 … Cg1^***ii***^ ****= 2.69 Å (where Cg1 is the ring centroid of ring C31 … C36 located at symmetry position ***ii* **= *x*, 3/2 − *y*, −1/2 + *z*) C22 … Cg1^***ii***^ ****= 3.4811(17) Å. The combination of both of these generates a stacked structure parallel to the *ac* (010) plane. The remaining contacts are at the van der Waals contact distances. The KPI packing index is 69.1 and this is reflected in the multitude of weak but cumulatively important C–H … O, C–H … π and π … π stacking interactions in the crystal structure.[26]

**Figure 5.**
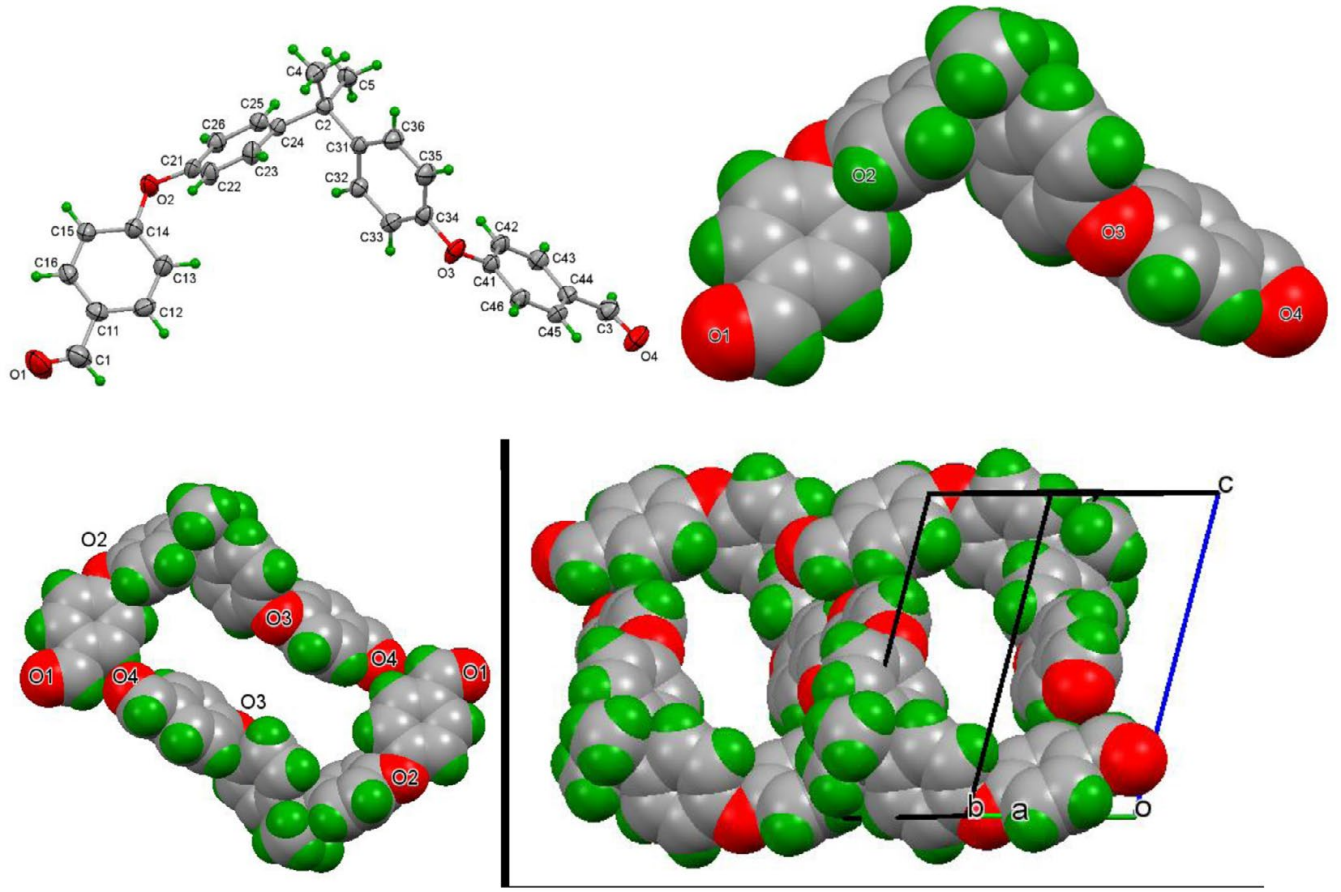
ORTEP diagram of **3e** with ellipsoids at the 30% probability level; similar orientation with atoms as van der Waals spheres; view of the primary hydrogen bonding interactions forming a hydrogen bonded ring and a view with chains of rings with unit cell*.*

Molecules of **3e** are non-planar by necessity as directly influenced by the *gem*-dimethyl group and the –C_6_H_4_–O–C_6_H_4_– linkages. The four aromatic rings are between angles of 32.15(16)° (for rings 1_C11_ and 4_C41_) and 81.76(9)° (rings 3_C31_ and 4_C41_) to one another although the stepwise inter-planar angles along rings 1 to 4 are close to being orthogonal at 62.85(6)°, 79.68(6)°, and 81.76(9)°. The asymmetric unit contains one molecule of **3e** and molecular aggregation involves weak C–H … O hydrogen bonding (especially involving O4) together with cumulatively important and numerous weak C–H … π and π … π stacking interactions, though not compared to **3a**. Molecules of **3e** adopt an open structure about the *gem*-dimethyl hinge as can be viewed in Figure 5 and aggregation can be considered with molecules forming hydrogen bonded dimers at O4 related by inversion centers in a loose *R* ^2^_2_(6) arrangement (C3–H3 … O4^***iii***^ ****= 3.350(3) Å). The remaining C12–H12 … O4^***iv***^ (C12 … O4^***iv***^ ****= 3.315(3) Å) interaction links pairs of dimers about inversion centers to form hydrogen bonded rings that ultimately gives rise to chains of rings along the *a*-axis direction and parallel with the (011) plane (symmetry codes ***iii* **= 4 − *x*,1 − *y*,1 − *z* and ***iv* **= 2 − *x*,1 − *y*,1 − *z*).

The KPI packing index is 66.5 which is substantially less than what would be expected in organic structures.[26] For example, in a series of ten organic structures with extensive amide … amide or amide … pyridine hydrogen bonding the packing indices range from 67.5 to 72.0 with an average of 70.0.[24] However, there are no solvent accessible voids in the lattice and the packing index is suggestive of a loosely packed structure with two primary but weak C–H … O intermolecular interactions and additional weak contacts. The molecules pack and stack rather loosely in the crystal structure. It may be of interest to examine how crystals of **3e** react with reactive di-functional species in a solid-state reaction given how close the di-aldehydes are in proximity to one another in the solid-state.

### 3.3. Syntheses of polymers

**Figure 6.**
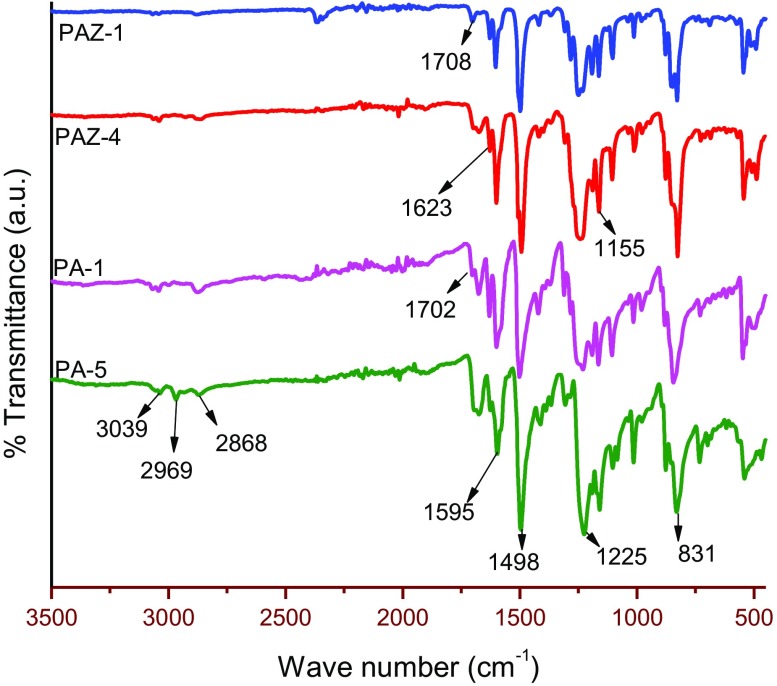
FT-IR spectra of polyazomethines*.*

**Figure 7.**
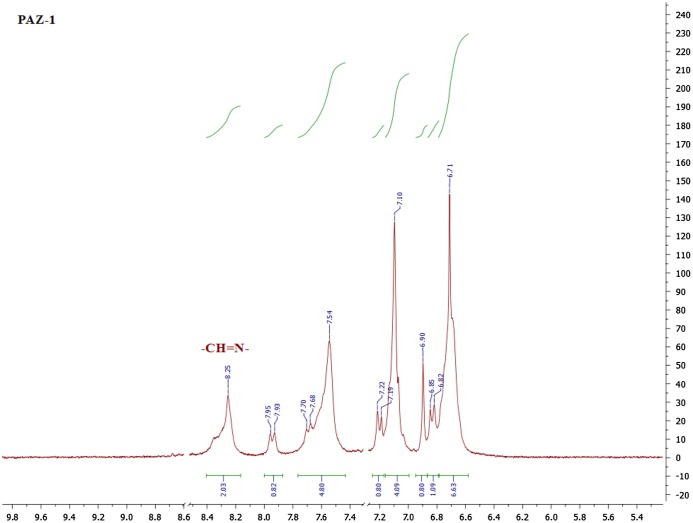
Proton NMR spectrum of PAZ-1*.*

The formation of PAM is confirmed by FT-IR and NMR spectroscopic measurements and the results are presented in Table 1. From comparison of FT-IR spectral data of monomers and polymers it can be clearly seen that the characteristic aldehydic (–CHO) peak in monomers at about 1692 to 1686 cm^−1^ has been disappeared in polymers, which is in accordance with literature.[15] The characteristic azomethine stretching bands occurring at 1620–1633 cm^−1^ (shown in Figure 6) accompanied with singlet at δ 8.25 ppm (shown in Figure 7) corroborate the formation of azomethine linkages. The smaller bands at 1702 and 1708 cm^−1^ for polymers PA-1 and PAZ-1 (see Figure 6) may be ascribed to aldehydic end groups of macrochains. Meanwhile, the bands assigned to PA-5 at 3039, 2969 and 2868 cm^−1^ are due to aromatic rings and aliphatic –C(CH_3_)_2_ groups, respectively. Likewise, the singlets appearing in the range of δ 8.23–8.44 ppm are because of –CH=N–, and remainder peaks (at δ 7.96–6.62 ppm) are assigned to aromatic protons or –C(CH_3_)_2_ protons (see Table 1).

**Table 1.** FT-IR and ^1^H NMR spectral data of polyazomethines.

Polymer	IR (KBr) cm^−1^	^1^H NMR (300 MHz, D_2_SO_4_, *δ* ppm) (s: singlet, m: multiplet)
PA-1	1633 (–CH=N–)	8.25 (s, azomethine), 7.94–6.71 (m, aromatic protons)
PA-3	1628 (–CH=N–)	8.33 (s, azomethine), 7.60–6.68 (m, aromatic protons)
PA-4	1620 (–CH=N–)	8.44 (s, azomethine), 7.62 (s, aromatic protons), 7.49–6.97 (m, aromatic protons), 6.73 (s, aromatic protons)
PA-5	1620 (–CH=N–)	8.31 (s, azomethine), 7.93–7.06 (m, aromatic protons), 2.29 (s, –CH_3_ protons)
PA-6	1621 (–CH=N–)	8.23 (s, azomethine), 7.72–6.76 (m, aromatic protons)
PAZ-1	1626 (–CH=N–)	8.25 (s, azomethine), 7.96–6.71 (m, aromatic protons)
PAZ-2	1627 (–CH=N–)	8.39 (s, azomethine), 7.82–6.62 (m, aromatic protons)
PAZ-4	1623 (–CH=N–)	8.37 (s, azomethine), 7.63–6.79 (m, aromatic protons)
PAZ-5	1621 (–CH=N–)	8.25 (s, azomethine), 7.82 (s, aromatic protons), 7.52–7.02 (aromatic protons), 1.76 (s, –CH_3_ protons)
PAZ-6	1626 (–CH=N–)	8.35 (s, azomethine), 7.92–6.73 (m, aromatic protons)

### 3.4. Organosolubility and inherent viscosities

The synthesized PAM were subjected to organsolubility studies. For this the solubility of PAM was determined qualitatively in several solvents by dissolving 10 mg of polymer powders in 1 mL of solvent at 30 °C. The solubility test results are shown in Table 2. The polymers showed a limited solvation behavior which may be attributed to high aromatic rings connected to each other that have caused packing of polymer chains, thus, giving rigidity, regularity, and symmetry in the backbone that resulted in lowering of solubility. Inter- and intra-molecular interactions that affected tight packing of polymer chains are responsible for lowering in solubility. The results show that the polymers are completely insoluble in non-polar solvents (i.e., *n*-heaxne) while increasing the polarity of solvent increased solubility. The strongest protic solvent, sulfuric acid, reduces/ or overcomes the chain-chain interactions by protonation of imine linkage and enhances solubility by penetrating into polyazomethine chains.

**Table 2.** Organosolubility and inherent viscosities of polyazomethines.

Polymer	*n*-hexane	CHCl_3_	DMF	DMSO	NMP	H_2_SO_4_	*η*_inh_(dl/g)
PA-1	− − h	− − h	+ − h	+ − h	+ − h	+ + +	1.76
PA-3	− − h	+ + +	+ − h	+ − h	+ − h	+ + +	1.93
PA-4	− − h	− − h	+ − h	+ − h	+ − h	+ + +	1.65
PA-5	− − h	+ − h	+ − h	+ − h	+ − h	+ + +	1.93
PA-6	− − h	+ + +	+ + +	+ + +	+ + +	+ + +	1.77
PAZ-1	− − h	− − h	− − h	− − h	− − h	+ + +	1.72
PAZ-2	− − h	+ − h	+ − h	+ − h	+ − h	+ + +	1.75
PAZ-4	− − h	− − h	− − h	− − h	− − h	+ + +	1.72
PAZ-5	− − h	+ − h	+ + +	+ + h	+ + h	+ + +	1.70
PAZ-6	− − h	+ + +	+ + h	+ + h	+ + h	+ + +	1.72

Notes:

− − h = insoluble on heating.

+ − h = partially soluble on heating.

+ + h = soluble on heating.

+ + + = soluble at room temperature.

The viscosity of PAM is a measure of resistance to cooperative segmental movement of macrochains. It depends on flexibility of macrochain, chain entanglement, inter- and intramolecuar interactions. The inherent viscosities (*η*_inh_) of ether-based aromatic PAM were determined in H_2_SO_4_ at 30 °C at polymer solutions with a concentration of 0.2 gdl^−1^ using U-tube Ubbelhode viscometer. The inherent viscosities were found to be in the range of 1.70–1.93 dlg^−1^ showing moderate to high molecular weight of polyazomethines. The higher value of inherent viscosity may also be attributed to rigidity of aromatic chains due to higher stacking efficiency.[30]

### 3.5. Thermal properties

**Figure 8.**
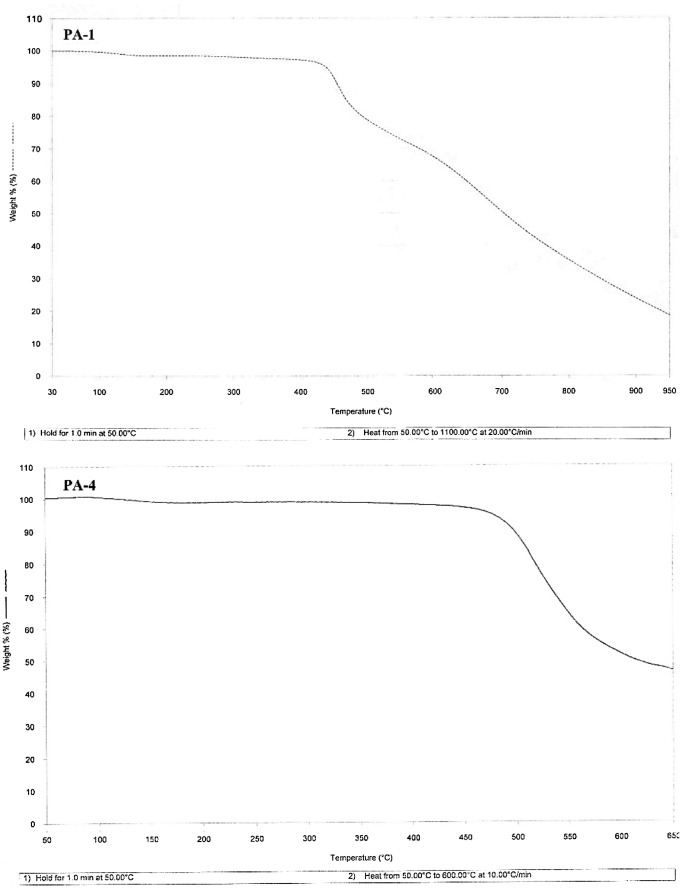
TGA curves of PA-1 and PA-4*.*

Thermal properties of PAM were investigated by DSC and thermogravimetric analysis (TGA) (Figure 8) and the data is presented in Table 3. Thermal data showed that the polymers are exceptionally thermally stable. The glass transition temperatures has not been observed in these polymers while they showed good thermal stability. The non-observation of glass transition temperatures suggest that polymers don’t undergo extensive cooperative segmental motions. This observation is concurrent to the organosolubility, powder XRD and supports the chain stiffness and packing efficiency of macrochains. The thermal behavior of these polymers has been shown in Table 3 in terms of *T*_on_, *T*_20%_, *T*_max_, *T*_F_ in degrees, and % char yield showing onset of degradation, 20% weight loss, maximum degradation temperature, and final degradation temperatures, respectively. The onset temperature of degradation (*T*_on_) lies from 142 to 450 °C with maximum stability to initial heating shown by polymers synthesized from monomer **3a** viz 420 and 450 °C. The maximum degradation temperature (*T*_max_) for PAM lies between the range 550 and 710 °C, only the polymer PAZ-6 showed lower maximum degradation temperature at 350 °C. The increased thermal stability can be accredited to rigidity of monomers and effective staking of aromatic moieties in macrochains.

**Table 3.** Thermal degradation data of polyazomethines.

Polymer	*T*_on_ (°C)	*T*_20%_ (°C)	*T*_max_ (°C)	*T*_F_ (°C)	% Char residue
PAZ-1	450	502	550	675	53
PA-1	420	495	650	950	18
PA-4	140	518	525	650	47
PAZ-5	142	400	700	950	24.5
PA-5	350	530	550	650	46.6
PAZ-6	180	450	350	600	60
PA-6	160	515	710	950	32

### 3.6. Powdered X-ray diffraction

**Figure 9.**
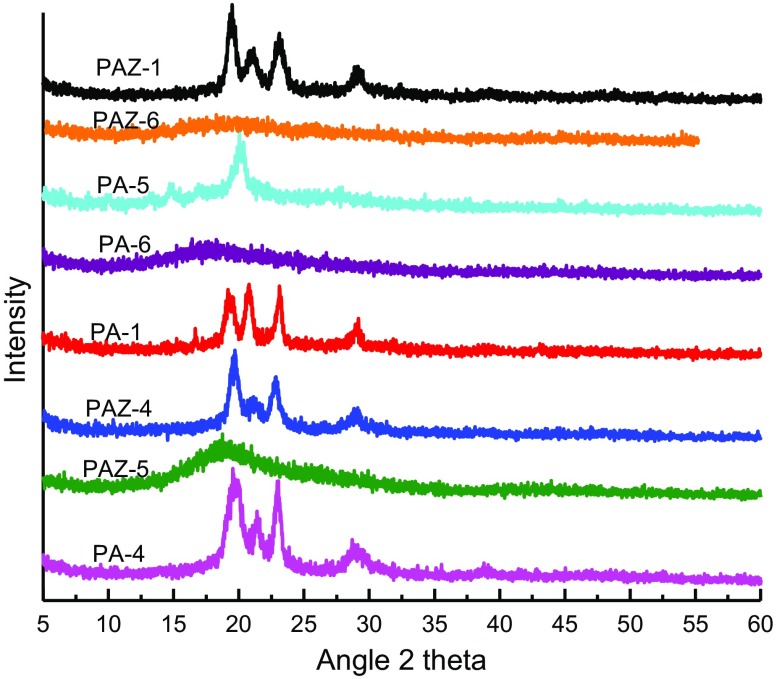
Powdered X-ray diffractions of polyazomethines*.*

The semicrystalline phases in PAM were examined with wide angle X-ray diffraction analysis and are presented in Figure 9. Irrespective of monomer used all the polymers showed semicrystallinity pattern in wade angle X-ray diffraction analysis (Figure 9). Only the polymers synthesized from monomers **3e** and **3f** that is PA-5, PA-6, PAZ-5, and PAZ-6 having –C(CH_3_)_2_ and –C(CF_3_)_2_ groups in backbone showed amorphous nature. This clearly indicates that the symmetry of polymer chain has a role in semicrystalline behavior. The kinky groups –C(CH_3_)_2_ and –C(CF_3_)_2_ in the backbone of polymers hinder in the close packing, thus rendering them amorphous.

### 3.7. Solvatochromism, acidochromism and PL

The optical properties and electronic states of the polymers were examined by UV–vis and PL measurements in the concentration range ~10^−5^ to 10^−8^ M solutions in CHCl_3_, DMF, and sulfuric acid. The results of photophysical spectral data are tabulated in Table 4 and shown in Figure 10.

**Table 4.** Photophysical spectral data of polyazomethines.

Polymer	*λ*_max_ (nm)^a^	*λ*_max_ (nm)^b^	*λ*_max_ (nm)^c^	*λ*_onset_ (nm)^c^	*E*_g_ (eV)^c^^,^^d^	PL *λ*_max_ (nm)^c^	Stoke’s shift (nm)
PA-1	–	–	426, 286	487	2.55	484	58
PA-3	356, 281	363, 287	399, 284	479	2.59	479	80
PA-4	–	–	418, 283	484	2.57	480	62
PA-5	368	–	405, 285	475	2.61	483	78
PA-6	361, 279	363, 283	408, 289	486	2.55	482	74
PAZ-1	–	–	399, 286	465	2.67	493	94
PAZ-2	–	337	426, 285	551	2.25	485	59
PAZ-4	–	332, 296	388, 284	465	2.67	484	96
PAZ-5	335, 283	336, 283	386, 283	471	2.64	482	96
PAZ-6	335, 280	335	392, 286	463	2.68	483	91

^a^ The absorbance measured in chloroform.

^b^ The absorbance measured in DMF.

^c^ The absorbance measured in sulfuric acid.

^d^ Calculated from the equation: *E*_g_ = 1242/*λ*_onset_.

Figure 10.(a): UV–vis spectra of polyazomethines in CHCl_3_, (b): UV–vis spectra of polyazomethines in DMF, (c): UV–vis spectra of polyazomethines in sulfuric acid, (d): Photoluminescence spectra of polyazomethines in acidic solution*.*

**Figure:**
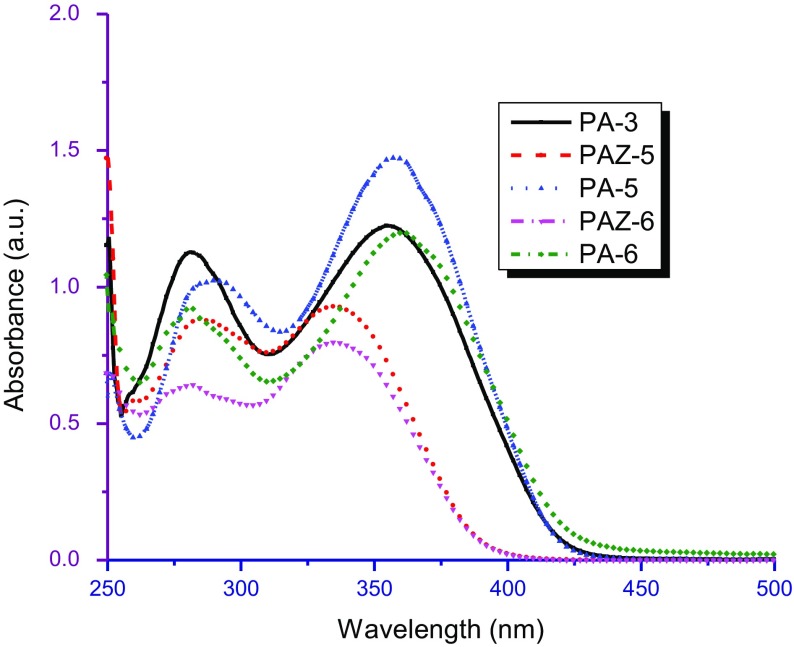

**Figure:**
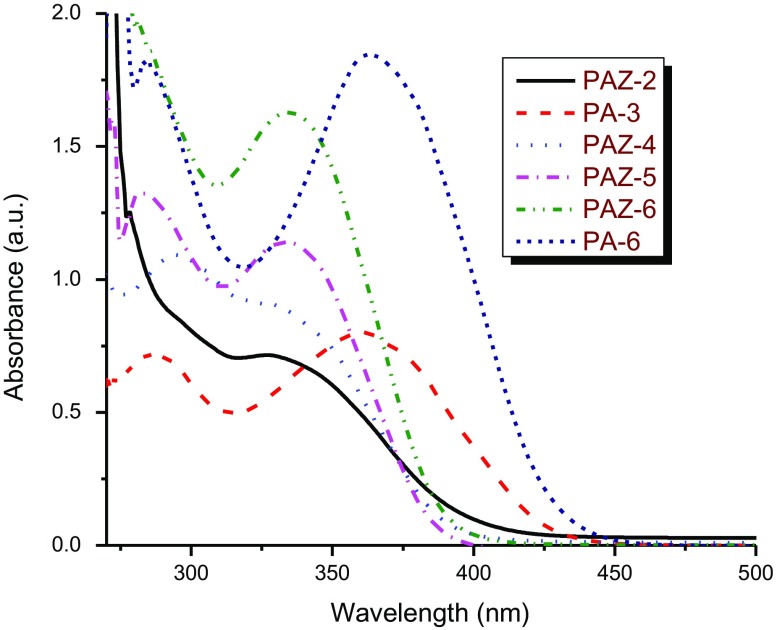

**Figure:**
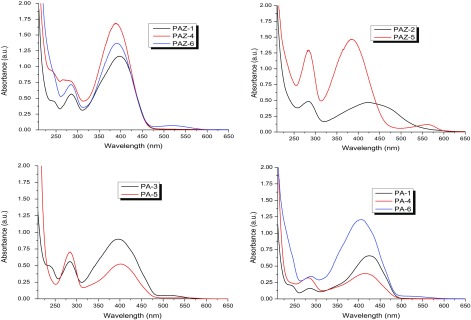

**Figure:**
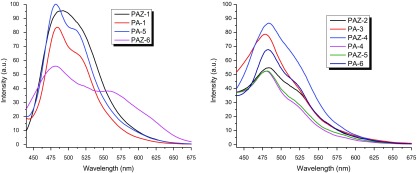

The UV–vis spectra of azomethines recorded at very dilute solutions gave maximum absorption at 335–368 nm in CHCl_3_ (Figure 10(a)) while 332–363 nm in DMF (Figure 10(b)). The solutions of partially soluble azomethines were filtered before taking electronic spectra. Completely insoluble azomethines PA-1, PA-4, and PAZ-1 showed no absorption bands in either solvent. The value of electronic absorption bands of PAM depend upon solvent polarity (dipole moment; *μ* = 1.01D for CHCl_3_, and *μ* = 3.86D for DMF). Therefore, absorption bands recorded in DMF overall showed red shift (positive solvatochromism) [30] which is believed to be due to enhanced interactions of polar sites of solvent with HOMO and LUMO levels of polymers.

Acidochromic behavior was viewed in UV–vis spectra of macromolecular azomethines (Figure 10(c)). The acidic solutions turned dark yellow or red while the corresponding neutral solutions were light colored. The drift in electronic absorption maxima of azomethines to bathochromic region infers the extension of delocalized *π*-conjugation length. Electronic spectra taken in sulfuric acid showed that the charge transfer from Bronsted acid to lone pair of electron on nitrogen in azomethine has taken place. It is believed that protonation of azomethine linkage resulted in coplanarity of polymer backbone, and thus led to increased π-electronic cloud delocalization over wider range which resulted in marked bathochromic shift.

PL measurements (Figure 10(d)) were conducted using 420 nm as excitation wavelength and emission spectra were recorded from 440 nm onward. All azomethines exhibited PL features and emitted blue light in the range of 480–493 nm. The highest PL intensity was found for PA-6 and PAZ-1 while lowest was observed for PA-4. Moreover, it was noted that the polymers of **3a**, **3e,** and **3f** monomer showed higher intensity as compared to other three monomers. The difference is energies of absorption maxima and emission maxima (called stokes shift) were indicted in terms of wavelength and occurred from 58 to 96 nm showing energy decline during electronic transitions. This energy loss of ~58–96 nm can be attributed to excimer formation as evidenced by bathochromic effect and emission bands enlargement (see Figure 10(d)).

### 3.8. Electrical conductivity

**Figure 11.**
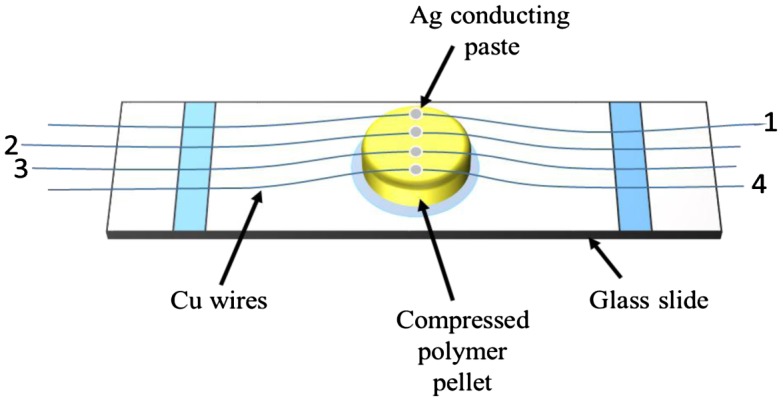
Schematic illustration of pelletized sample prepared for electrical conductance measurement*.*

**Figure 12.**
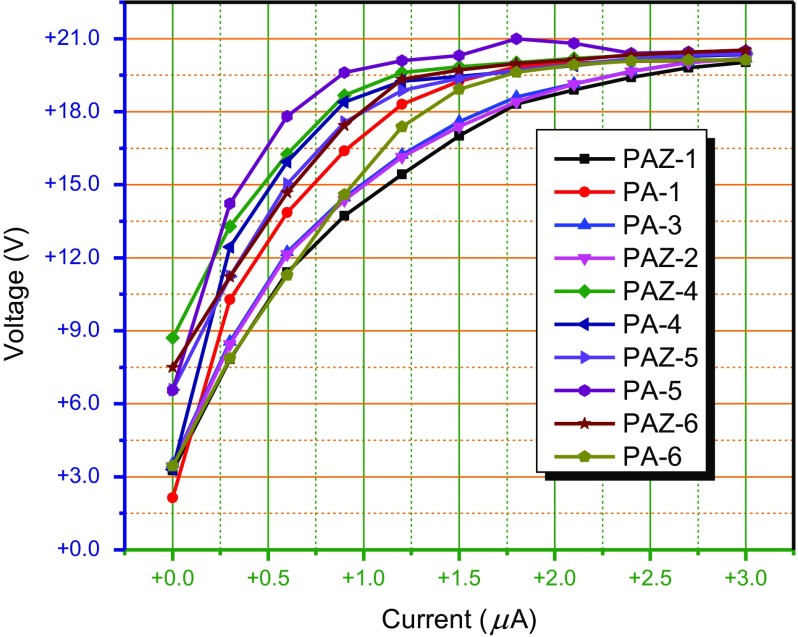
I–V curves of polyazomethines*.*

The solid state electrical conductivities of synthesized PAM were measured with Keithley-2400 source meter using pelletized powdered polymer samples. The pallets were prepared by pressing powdered sample at 4.9 metric ton pressure for 5 min. Then the conductance were measured using four probe technique as discussed above and depicted in Figure 11. Electrical connections were made with Cu wires, that were abraded with fine grit silicon carbide sandpaper, placed equidistant on pellet, and the Cu connections on pellet were fixed with silver conducting paste. The current (ranging 1–3 μA) was forced through the two points of probe designated as 1 and 4 (see Figure 11) while measuring the voltage (V) as output signal through points 2 and 3. Afterward calculated the conductance, and conductivities of polymers from slop of *I*-*V* curves (with pellet dimensions as: diameter = 1.25 cm; radius (*r*) = 0.625 cm; area (*A*) = 1.22718 cm^2^; and distance between two probes (*L*) = 0.3 cm) (Table 5 and Figure 12).

**Table 5.** Slop of I–V curves for the polyazomethines and subsequent calculations obtained by four probe method.

Polymer	Resistance (*R*) × 10^3^ Ω	Conductance (*G*) × 10^−3^ Ohm^−1^	Conductivity (*σ*) × 10^−3^ Scm^−1^
PA-1	4.74437 ± 1.07	0.21	0.051
PA-3	4.94471 ± 0.73	0.20	0.049
PA-4	12.61553 ± 2.99	0.08	0.019
PA-5	8.40298 ± 2.24	0.12	0.029
PA-6	9.13482 ± 0.90	0.11	0.026
PAZ-1	5.12437 ± 0.69	0.20	0.049
PAZ-2	4.97958 ± 0.72	0.20	0.049
PAZ-4	9.05963 ± 1.28	0.11	0.026
PAZ-5	10.30877 ± 1.29	0.10	0.024
PAZ-6	8.40195 ± 1.01	0.12	0.029

The measured electrical conductivities of pelletized powder are in the range of 0.019–0.051 mScm^−1^ which are many folds higher than the conductivities of any undoped common polyazomethine.[5,18,31]

The PA-1 has highest conductivity which is likely due to the straight pathway of electronic conduction provided by 3a and 4a. The 3a monomer have three benzene rings linked at para-position to each other and the monomer 4a also have two –NH_2_ groups linked at para positions. This configuration gives a straight chain polymer, and hence highest conduction.[32]

The polymer PA-4 synthesized from 4a have, in contrast to PA-1, lowest conductivity. The lowest conductivity is possibly due to the biphenyl moiety of the 3d monomer. In 3d the two benzene rings of biphenyl are tilted perpendicular to each other due to steric hindrance, which contributes to lagging conductivity value. This situation is more aggravated by *p*-phenylene diamine which forms two rigid C=N linkages tilted at both sides of the benzene ring at an angle of approximately 120 °, since the nitrogen atom being sp^2^ hybridized.[33]

The similar conductivities, and close to highest value, of polymers PA-3, PAZ-1 and PAZ-2, are possibly due to ortho, para, and meta-electronic effects. The intermediate, and close to each other, electrical conductivities of PA-5, PA-6, PAZ-5, and PAZ-6 show that the effect of break in conjugation due –C(CH_3_)_2_– and –C(CF_3_)_2_– is comparable to biphenyl ring effect of 3d. This is why the polymer PAZ-4 has also similar conductivity. Overall the conductivities of polymers synthesized from 4a have more conductivity than the polymers from 4b. The lower conductivities of 4b polymers possibly be related to ether-linkage between two benzene rings.

Certainly better quality data of conductivities are obtained for single-crystal samples using four-probe techniques, but these measurements are often not possible to perform, as polymeric materials can be obtained only in the form of microcrystalline powders. Therefore, rapid evaluation of electrical conductivities at room-temperature can be done with small quantities of any polymeric and/or solid material that can be compressed into a small pellet. This is due to the anisotropic property of the conductivity exhibited by nearly all ‘organic metals’ and semiconductors, that is the electrical conductivity, *σ*, value varies along different crystal axes. Obviously, a compressed pellet will comprise random spatial orientations of the polymeric microcrystals. Hence, the bulk conductivity of the pellet is a weighted average value measured largely by the value of *σ* along the least conducting axis of the crystallite sites and by inter-particle resistance. It must be noted that the conductivity values of organic polymers in the form of compressed pellets are typically 1–3 orders of magnitude lower than the same measured in the form of single crystals. In spite of the fact, the reliable data can be obtained on compressed polymer pellets because conductivity is determined mainly by short-range order, not long range order which is present only in single crystals.[19]

## 4. Conclusions

We deduce that the solubility of these polymers may be a slight hindrance to their application but they are very stable and their electrical conductivity is such a favorable property that tailoring them for physicochemical properties through introduction of long chain aliphatic substituents to aromatic rings of the main chain can lead to increased solubility and therefore processability. As such, these polymers with modified substituents can be potentially useful for optoelectronic and semiconducting applications. The blue light emitting ability 480–493 nm of azomethines is for consideration in the fabrication of blue light emitting devices. The electrical conductivities of these polymers are many fold higher than reported values for undoped azomethines.[34–36] Therefore, if these polymers are doped with any material such as an acid or metal ion which can cause drift in the azomethine electrons, thus providing conduction pathway to electron across the chain and/between the chain through electron hopping, then it is to be hoped that their conductivities can be increased by several orders of magnitude.

## Disclosure statement

No potential conflict of interest was reported by the authors.

## Funding

The authors are grateful to the Higher Education Commission of Pakistan for financial support under research project No. 20-2154/NRPU/R&D/HEC/12-3698.

## Supplementary data

The supplementary material for this paper is available online at http://dx.doi.org/10.1080/15685551.2016.1231042

## Supplementary material

Electronic Supplementary Information (ESI) available: The spectroscopic and single crystal data for the two isomers are provided with CCDC reference codes 1468964 and 1468965**.** Copies available, Email:deposit@ccdc.cam.ac.uk. This information is available free of charge via the internet.

## Supplementary Material

TDMP_1231042_Supplementary_Material.pdf
