# Supplementary material for: Liquid phase synthesis of aromatic poly(azomethine)s, their physicochemical properties, and measurement of ex situ electrical conductivity of pelletized powdered samples
Source: Des Monomers Polym. 2016 Sep 23;20(1):74–88. doi: 10.1080/15685551.2016.1231042 (PMC5812128; doi:10.1080/15685551.2016.1231042)

Electronic Supplementary Information

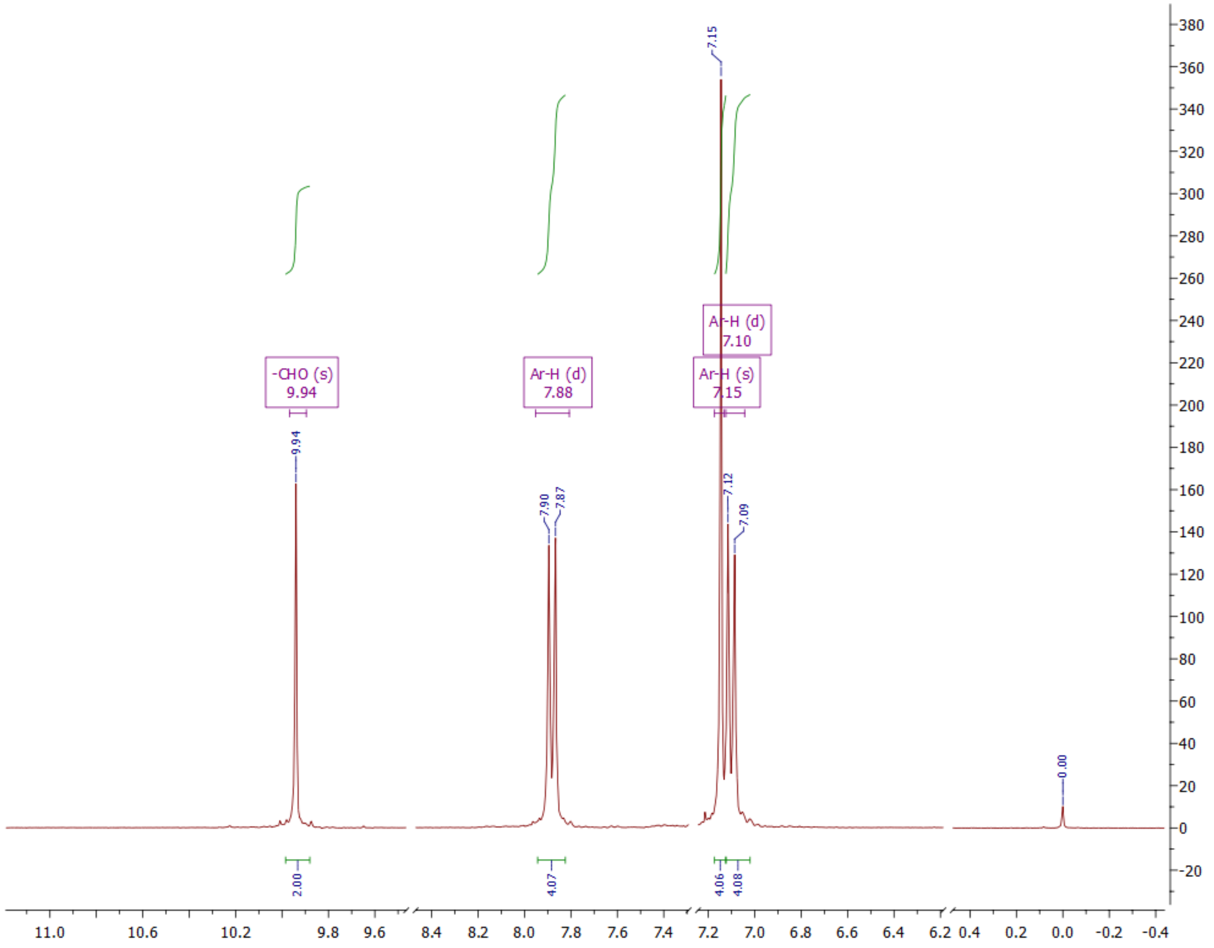

Figure 1: H MNR of 3a

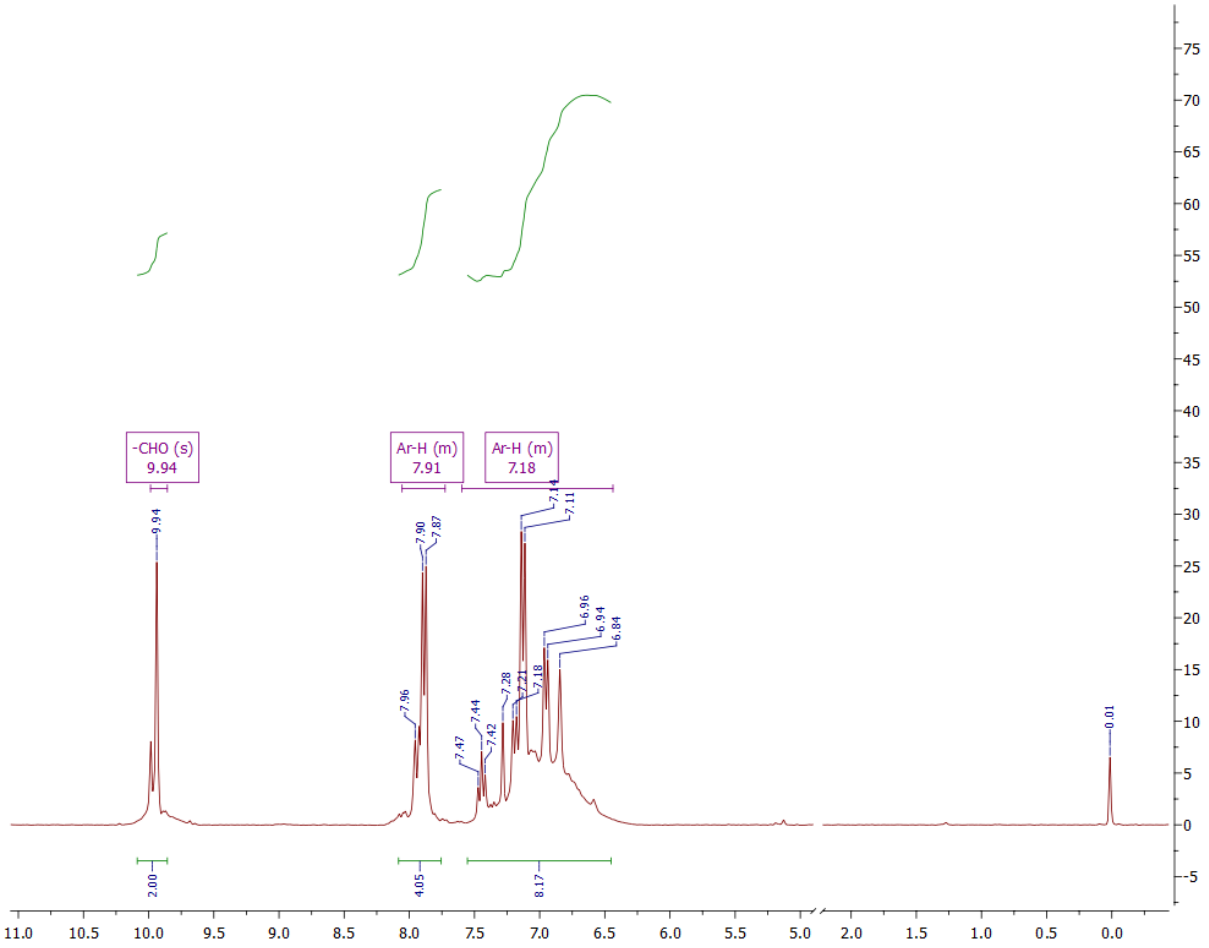

Figure 2: H NMR of 3b

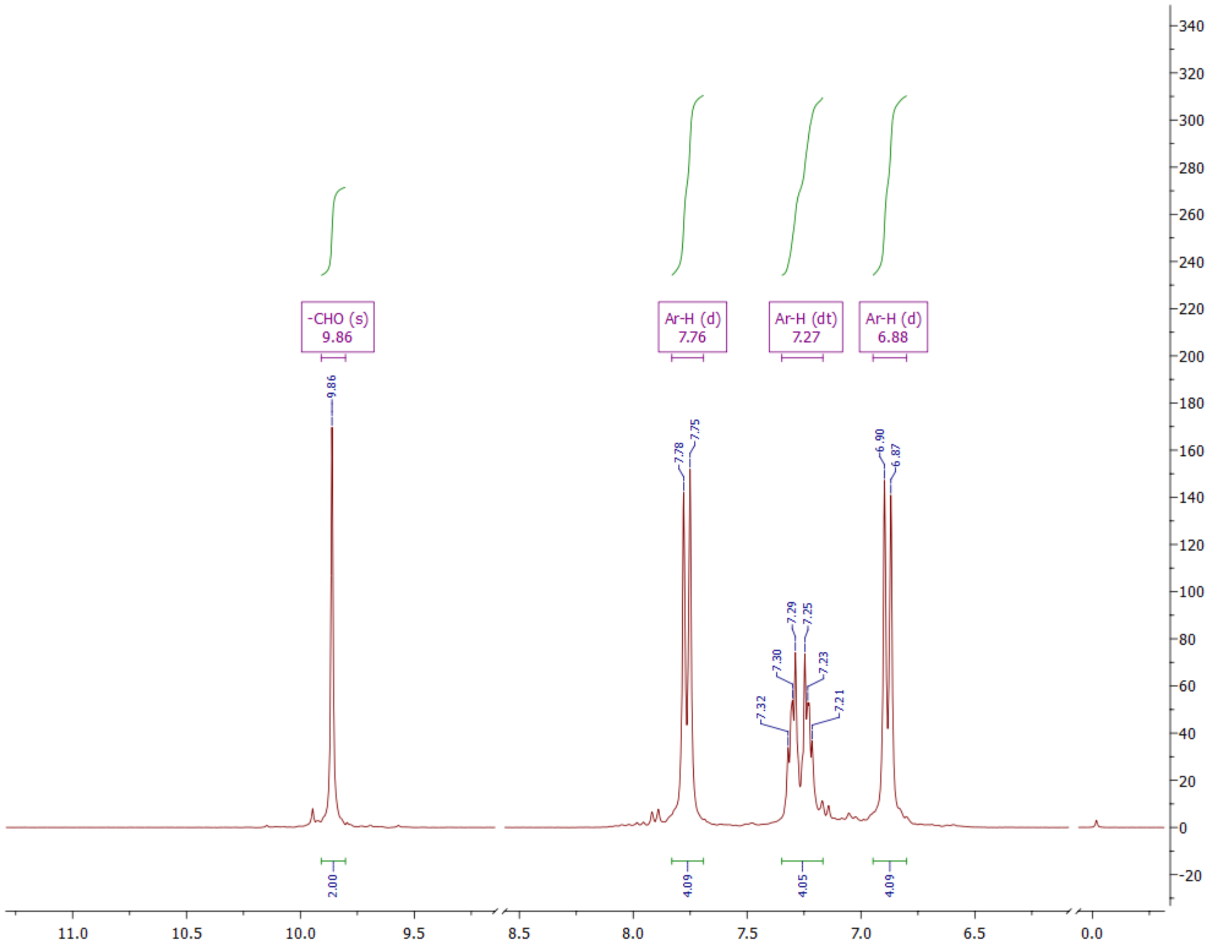

Figure 3: H NMR of 3c

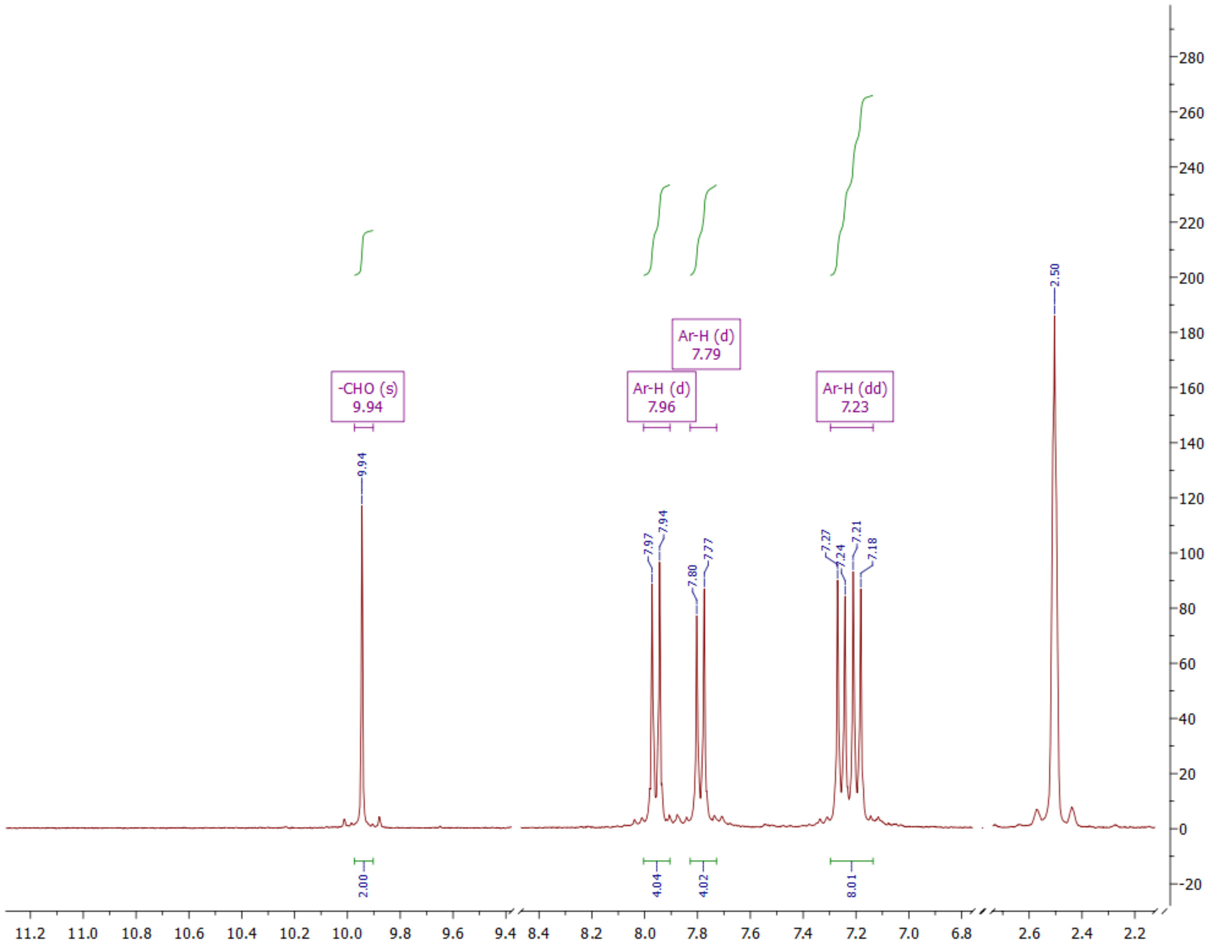

Figure 4: H NMR of 3d

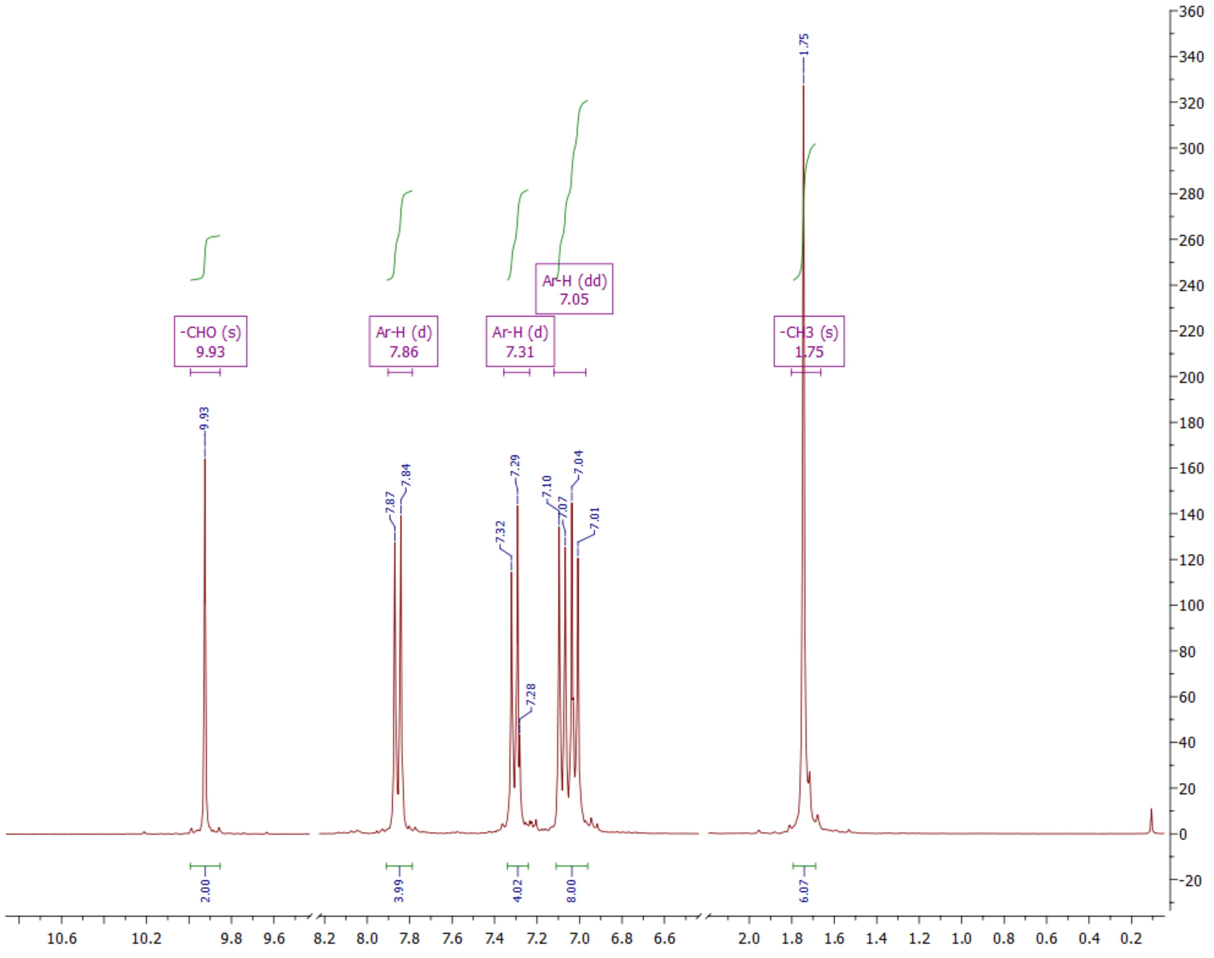

Figure 5: H NMR of 3e

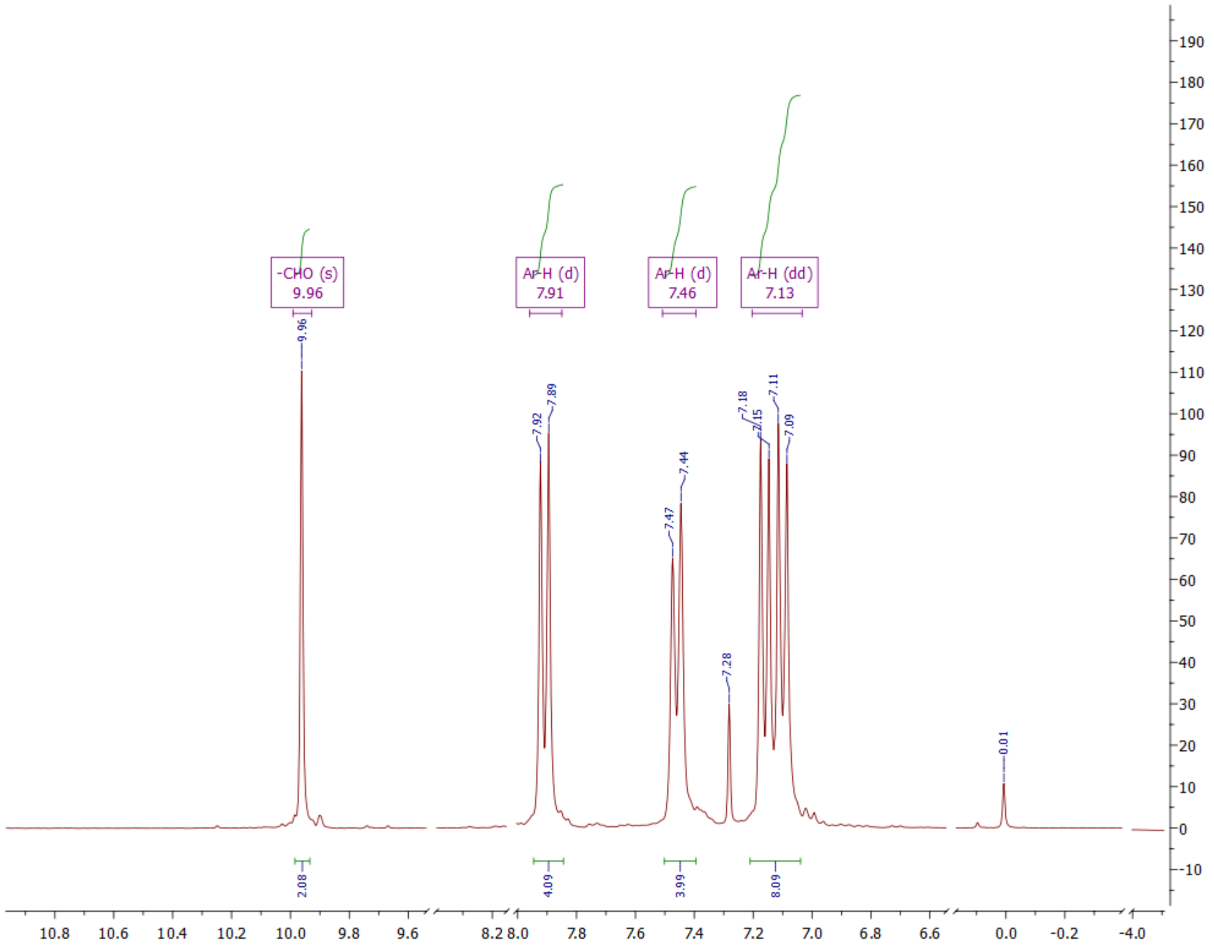

Figure 6: H NMR of 3f

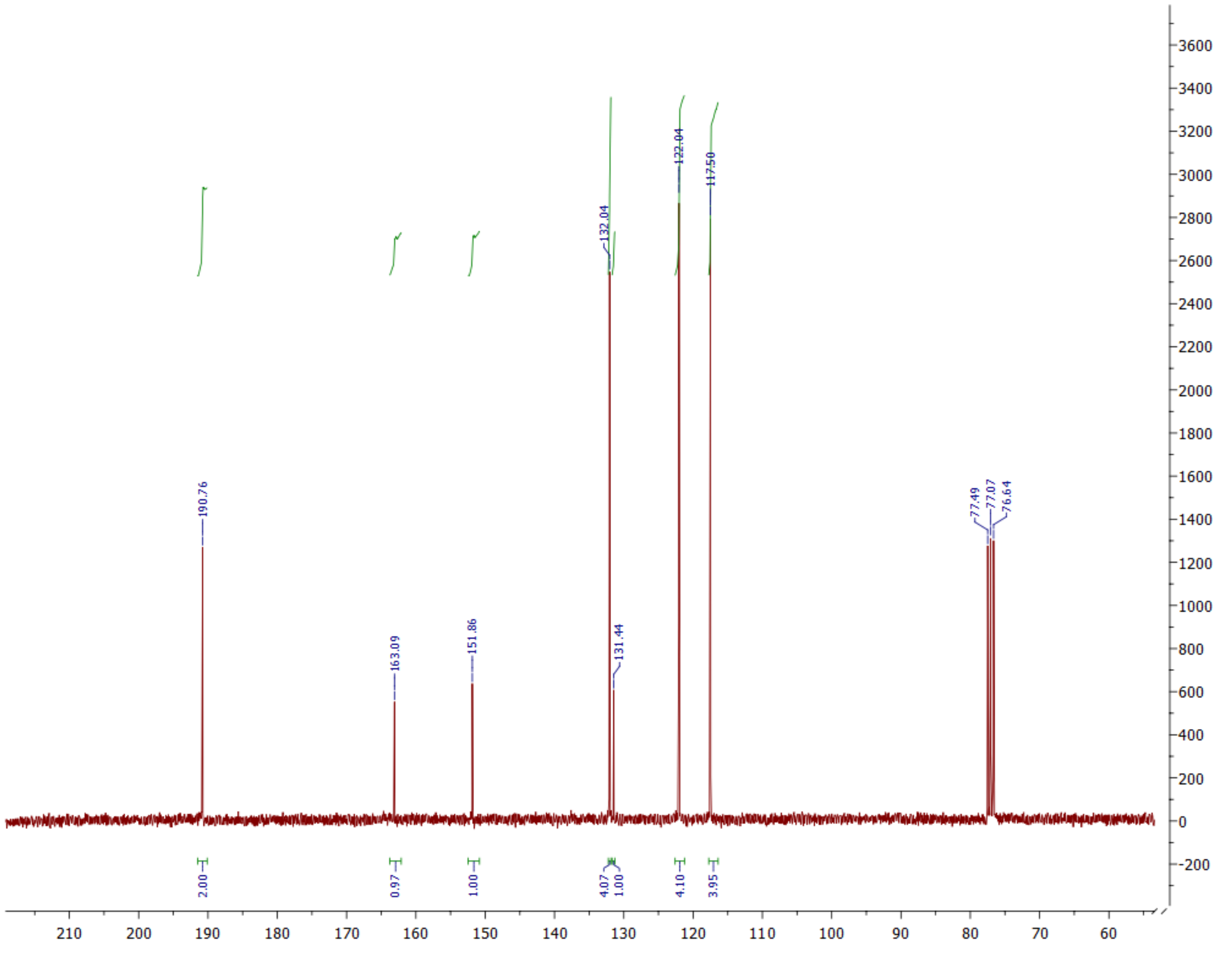

Figure 7: ^{13}C NMR of 3a

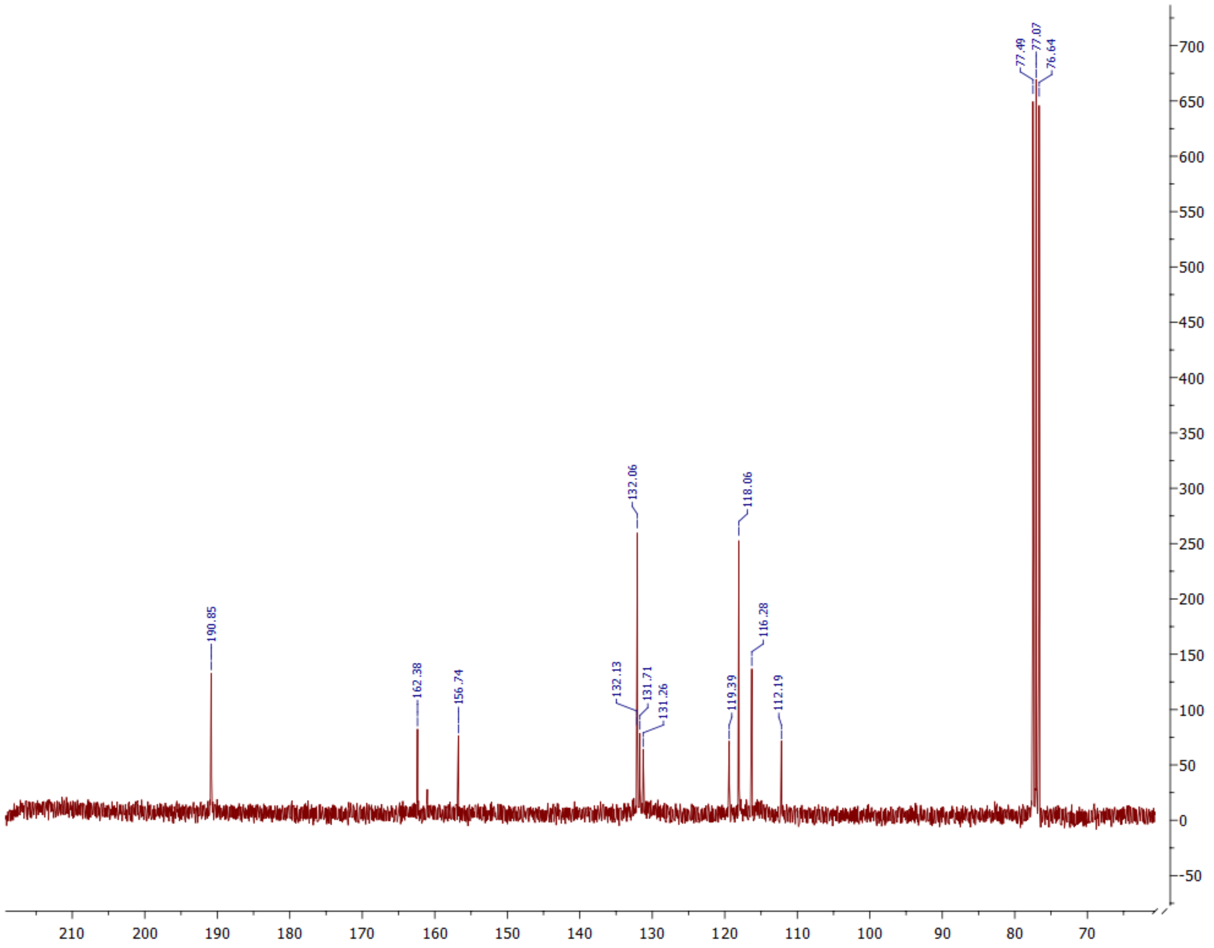

Figure 8: ^{13}C NMR of 3b

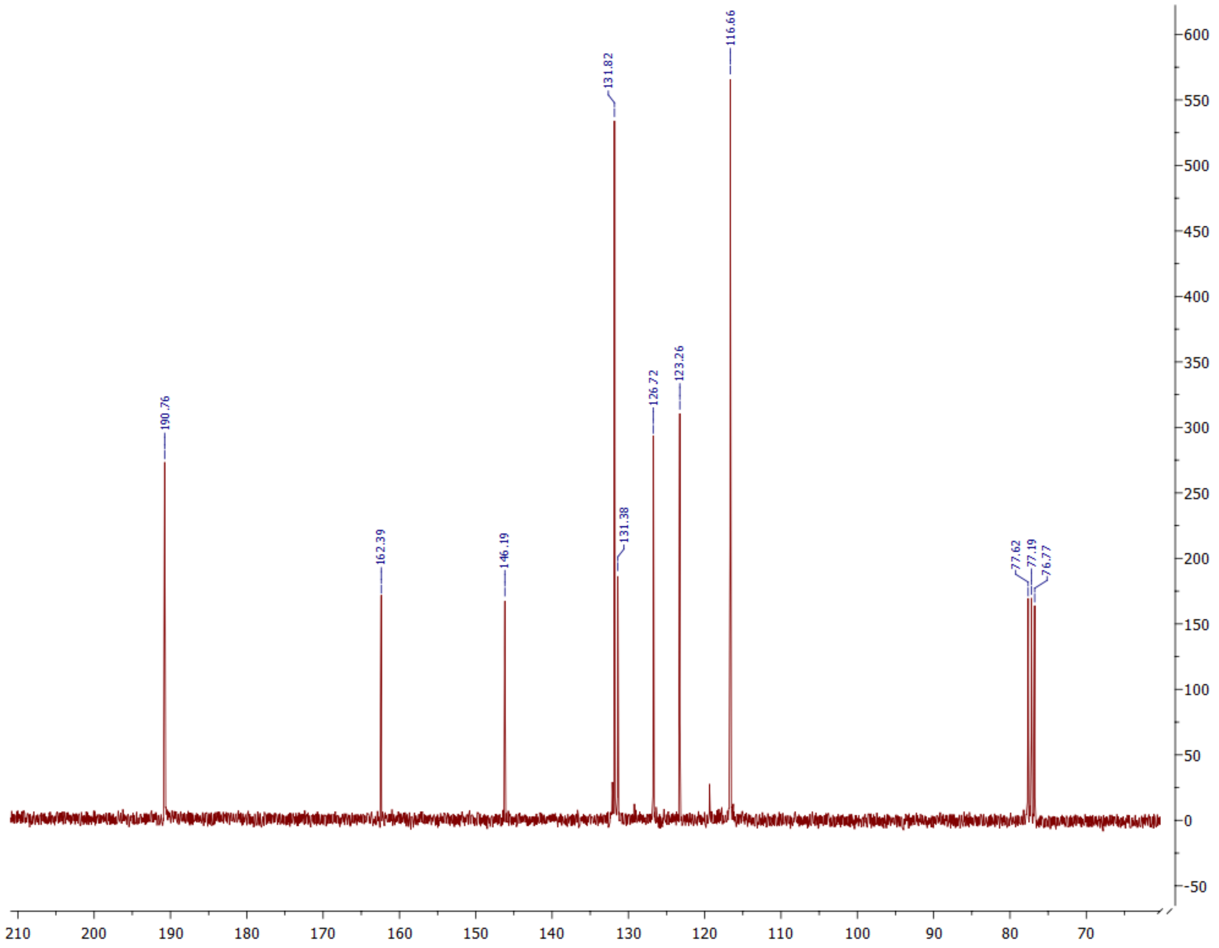

Figure 9: ^{13}C NMR of 3c

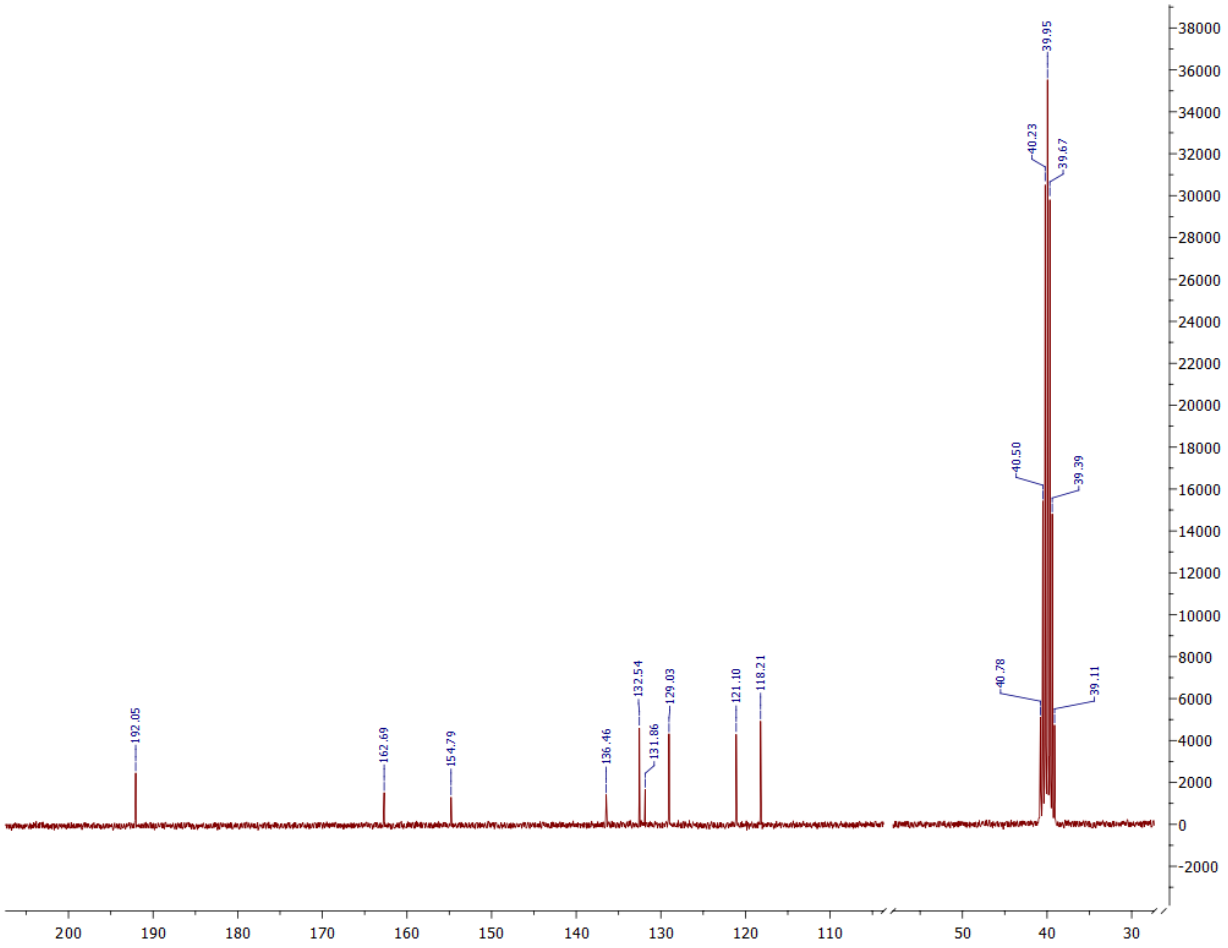

Figure 10: ^{13}C NMR of 3d

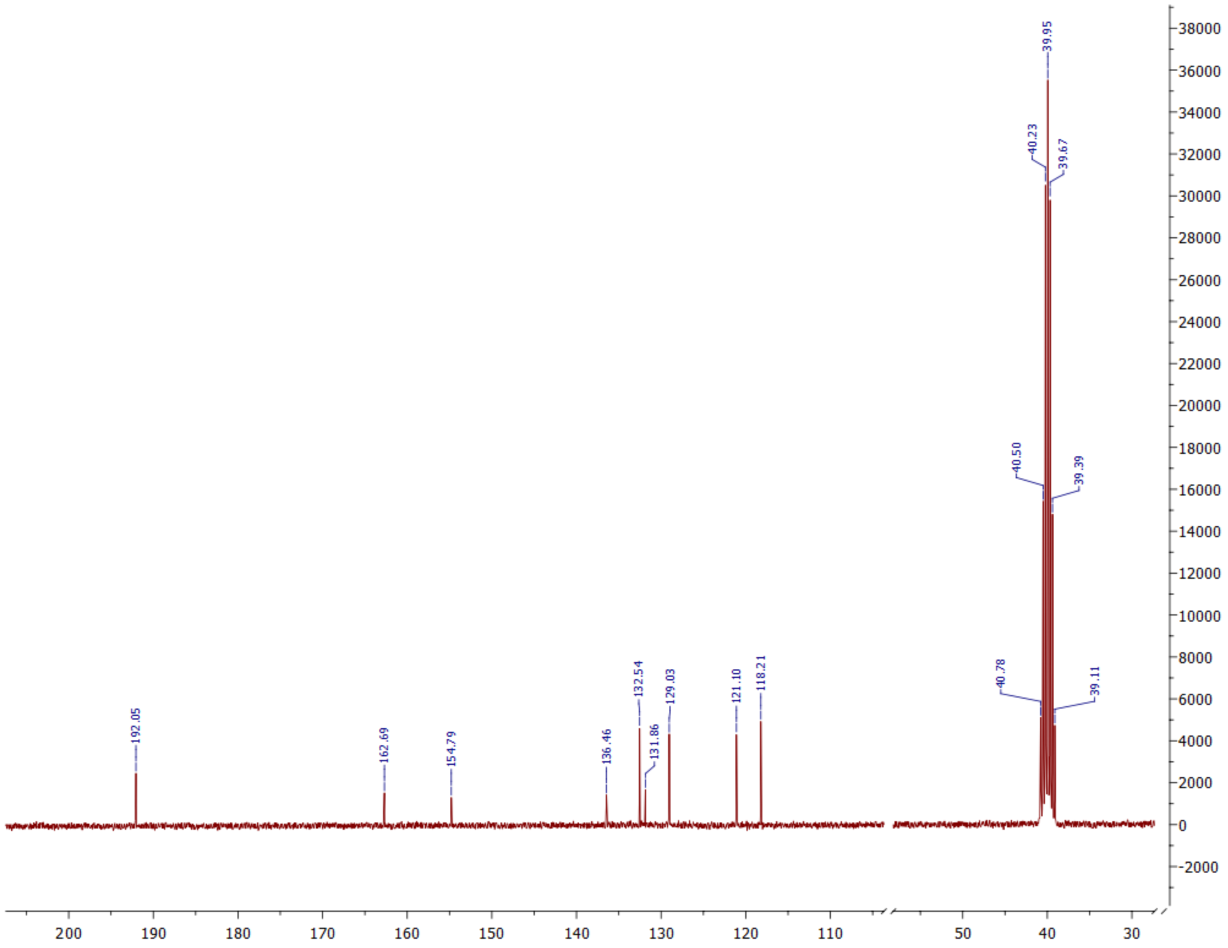

Figure 11: ^{13}C NMR of 3e

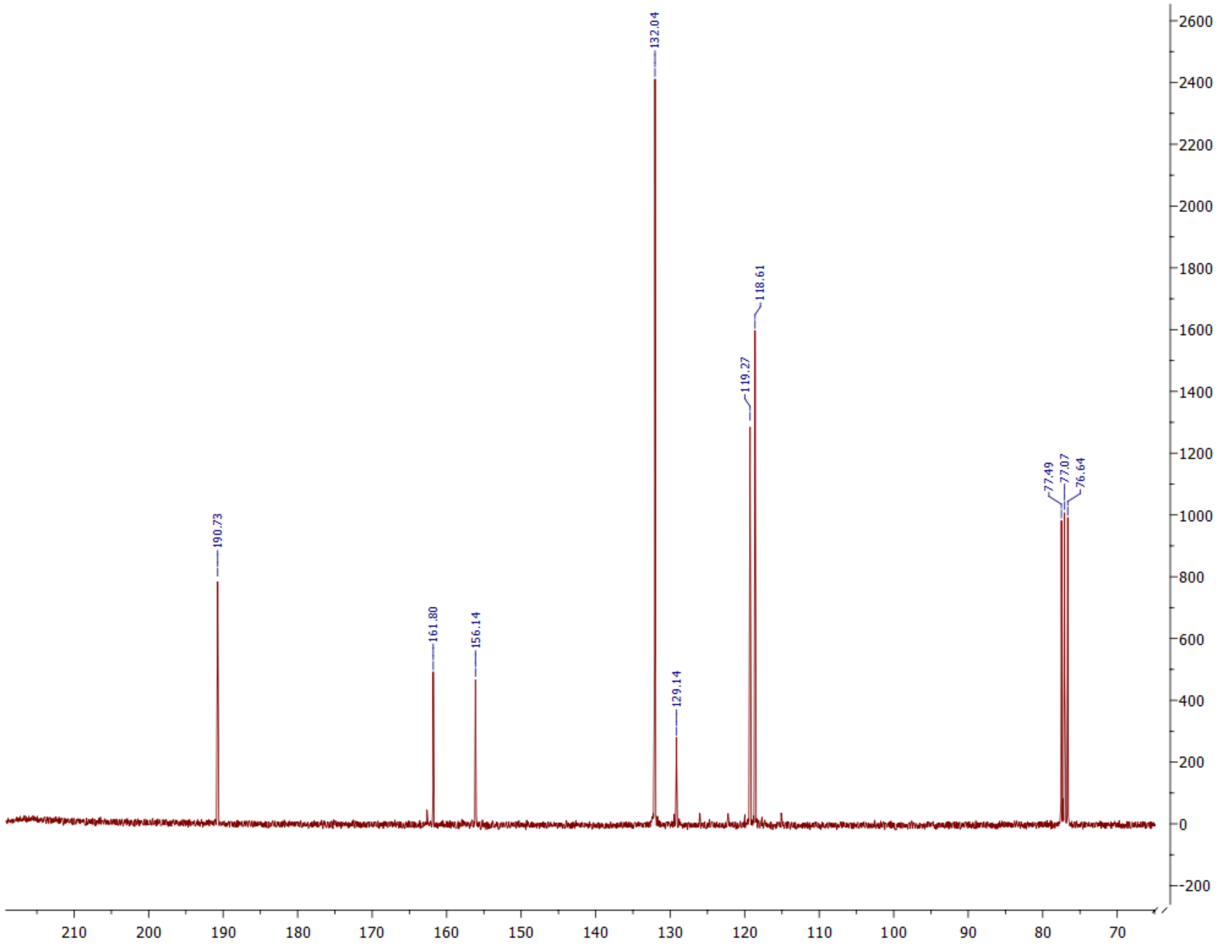

Figure 12: ^{13}C NMR of 3f

Table 1. Experimental Details of Single Crystals

Experiments were carried out at 294 K with Mo *K*α radiation using a Xcalibur, Sapphire3, Gemini Ultra. H-atom parameters were constrained.

	**3a**	**3e**
**Crystal data**		
Chemical formula	$C_{20}H_{14}O_4$	$C_{29}H_{24}O_4$
M_r	318.31	436.48
Crystal system, space group	Monoclinic, $P2_1/c$	Triclinic, $P\bar{1}$ (No.2)
a, *b*, *c* (Å)	9.7949(4), 10.6069(5), 15.1758(6)	7.5560(9), 11.762(2), 13.8277(18)
α, β, γ (°)	90, 97.783(4), 90	95.137(12), 103.256(10), 102.373(12)
V (Å^3)	1562.14(12)	1155.9(3)

Z	4	2
μ (mm^{-1})	0.09	0.08
Crystal size (mm)	0.47 × 0.41 × 0.14	0.65 × 0.55 × 0.40
**Data collection**		
Absorption correction	Analytical (ABSFAC, Clark & Reid, 1998)	Multi-scan; ABSPACK
T_{min}, T_{max}	0.964, 0.990	0.915, 1.000
No. of measured, independent and observed $[I > 2\sigma(I)]$ reflections	11810, 3465, 2443	11188, 6002, 3571
R_{int}	0.044	0.036
$(\sin \theta/\lambda)_{max}$ ($Å^{-1}$)	0.660	0.694
**Refinement**		
$R[F^2 > 2\sigma(F^2)]$, $wR(F^2)$, S	0.047, 0.129, 1.05	0.055, 0.154, 1.02
No. of reflections	3465	6002
No. of parameters	218	300
$\Delta\rangle_{max}$, $\Delta\rangle_{min}$ (e $Å^{-3}$)	0.18, -0.17	0.21, -0.16

Computer programs: *CrysAlis PRO*, Agilent Technologies, Version 1.171.34.49 (release 20-01-2011 CrysAlis171 .NET) (compiled Jan 20 2011, 15:58:25), *SHELXS14*/6 (Sheldrick, 2014), *SHELXL97* (Sheldrick, 2008) and SORTX (McArdle, 1995), *SHELXL2014*/7 (Sheldrick, 2014), *PLATON* (Spek, 2009), *SHELXL14*/6.

Table 2. Selected hydrogen-bond parameters

D—H···*A*	*D*—H (Å)	H···*A* (Å)	*D*···*A* (Å)	*D*—H···*A* (°)
**3a**				
C26—H26···O4^{i}	0.93	2.39	3.318 (2)	172
C22—H22···C35^{ii}	0.93	2.89	3.630(2)	137
**3e**				
C3—H3···O4^{iii}	0.93	2.57	3.351 (3)	142
C12—H12···O4^{iv}	0.93	2.56	3.315 (3)	139

Symmetry code(s): (***i***) *x*-1, -*y*+3/2, *z*-1/2; (***ii***) x, 3/2-y, -1/2+z (***iii***) -*x*+4, -*y*+1, -*z*+1; (***iv***) -*x*+2, -*y*+1, -*z*+1.

PLATON ckf file===

PLATON/ASYM-(Version 70316)-[Mode=2] FCF-File Validation for:15-008_3a

===

For Documentation: http://http://www.platonsoft.nl/FCF-VALIDATION.pdf

Section 1

===

General Data

===

Crystal Data From: platon.cif
Fo/Fc Data From: platon.hkl FCF-TYPE=LIST4
Space Group : P21/c
Wavelength (Ang) : 0.71073
Unit Cell (CIF) : 9.7949 10.6069 15.1758 90.000 97.783 90.000
SHELX WGHT Pars. : 0.0548 0.1507
Extinction Par. : 0.0097

Section 2

===

Reflections with abs((I(obs) - I(calc)) / SigW(I) > 3.0 [I(calc) from FCF]

===

Nr	H	K	L	Theta	I(obs)	I(calc)	Sigma(I)	Ratio	SigW(I)	RatioW
1	1	6	0	11.79	369.90	285.15	11.42	7.42	21.74	3.90
2	3	5	1	11.74	210.44	257.20	5.28	-8.86	15.48	-3.02
3	-1	6	1	11.84	54.59	36.70	4.11	4.35	5.37	3.33
4	1	6	1	11.90	20.11	32.11	2.08	-5.77	3.31	-3.63
5	-1	6	2	12.04	35.83	50.08	2.55	-5.59	4.42	-3.23
6	-10	7	3	25.63	-1.60	5.76	2.20	-3.35	2.34	-3.15
7	1	0	4	6.08	68.29	52.64	2.48	6.31	4.99	3.13
8	-10	5	4	23.71	4.51	14.32	2.09	-4.69	2.53	-3.88
9	-1	6	4	12.89	55.79	73.24	3.13	-5.58	5.80	-3.01
10	4	7	7	19.41	10.84	22.07	3.06	-3.67	3.62	-3.10

11	-4	0	8	12.88	61.05	90.62	3.96	-7.47	6.89	-4.29
12	-1	0	8	10.82	22.86	35.10	2.10	-5.83	3.46	-3.54
13	3	5	10	18.72	74.75	103.75	4.77	-6.08	7.97	-3.64
14	5	5	11	22.14	47.22	29.34	5.07	3.53	5.90	3.03
15	-5	4	12	20.08	3.68	11.97	1.87	-4.43	2.27	-3.66
16	-4	10	12	26.77	3.43	10.39	1.94	-3.59	2.27	-3.06
17	-8	2	13	23.60	0.14	7.44	2.06	-3.54	2.25	-3.24
18	4	4	15	25.04	-0.52	4.64	1.17	-4.41	1.37	-3.78
19	4	1	16	25.15	26.46	10.81	4.59	3.41	4.92	3.18
								--------		--------
							Average =	-2.52		-1.67

Note: SigW(I) is the SHELXL optimized weight

For I(calc) < 2 Sigma(I): = 2.61 and = 1.93

Section 3
==
Missing Reflections (Asym. Refl. Unit) below sin(th)/lambda = 0.5
==

Nr	H	K	L	sin(th)/lambda	Theta	I(calc)	I(calc)/I(max)
1	1	0	0	0.052	2.10*	441.79	0.00903
2	0	1	1	0.058	2.35*	2.01	0.00004
3	0	0	2	0.067	2.71*	488.47	0.00998

** Note: I(max) is the maximum I(obs) encountered in the fcf-file **

Starred Reflections have a Theta below Theta(Min) = 2.84

From CIF: Theta(Min) = 2.85

Section 4:
==

Resolution & Completeness Statistics (Cumulative and Friedel Pairs Averaged)

===

Theta	sin(th)/Lambda	Complete	Expected	Measured	Missing
20.82	0.500	0.998	1638	1635	3
23.01	0.550	0.999	2181	2178	3
25.24	0.600	0.999	2833	2830	3
------------------------- ACTA Min. Res. ---					
27.51	0.650	0.962	3584	3448	136
27.96	0.660	0.924	3749	3465	284

Note: The Reported Completeness refers to the Actual H,K,L Index Range

Section 5

===

R-Value Statistics as a Function of Resolution (in Resolution Shell)

===

Theta	sin(Th)/L	#	R1	wR2	S	Rs	av(I/SigW)	av(I)	av(SigW)
12.38	0.302	354	0.031	0.086	1.194	0.017	13.12	1117.70	65.25
15.68	0.380	359	0.038	0.103	1.083	0.037	9.24	169.01	12.03
18.02	0.435	367	0.032	0.085	0.844	0.038	8.56	175.49	12.74
19.90	0.479	351	0.046	0.126	1.069	0.057	7.04	100.47	8.79
21.51	0.516	354	0.052	0.144	0.978	0.077	5.37	66.35	7.07
22.94	0.548	371	0.067	0.172	0.947	0.119	4.27	36.64	5.31
24.22	0.577	361	0.089	0.235	0.980	0.190	3.14	19.45	4.17
25.40	0.603	357	0.110	0.301	1.027	0.252	2.53	14.96	4.10
26.49	0.628	353	0.117	0.337	0.996	0.319	2.11	11.05	3.78
27.52	0.650	222	0.132	0.380	0.944	0.399	1.72	11.29	4.71
27.96	0.660	16	0.083	0.509	0.959	0.544	1.32	8.29	4.66

R(sig) = sum(sig(I)) / sum(I) = 0.0357

```
------------------------------------------------------------------------------
------
From FCF: R1 = 0.0474(   2442), wR2 = 0.1292(   3465), S =   1.045
From CIF: R1 = 0.0474(   2443), wR2 = 0.1292(   3465), S =   1.045, Npar
=  218

No (SHELXL) Optimized Weights:  wR2 = 0.0866         , S =    1.64

Section 6
==============================================================================
======
Summary of Reflection Data in FCF - Note: Friedel Pairs Averaged
==============================================================================
======
Total # of Reflections in FCF.   3465  (Hmax = 12, Kmax = 13, Lmax = 19)
Obs
Number above Rep. Theta(Max) .      1
Actual   Theta(Max) (Deg.) ... 27.962  (Hmax = 12, Kmax = 13, Lmax = 19)
Exp
Reported Theta(Max) (Deg.) ... 27.962  (Hmax = 12, Kmax = 13, Lmax = 19)
Rep
Actual   Theta(Min) (Deg.) ...  2.845
Reported Theta(Min) (Deg.) ...  2.845

Unique (Expected) ...........   3748
Unique (in FCF) .............   3465
Observed [I .gt. 2 Sig(I)] ...   2444
Less-Thans ..................   1021
Negative Intensities .........    217
Negative Intensities < - 2 SIG      0

Missing (Total) .............    284
Missing Below Th(Min) ........      3
Missing Th(Min) to STh/L=0.600      0
Missing STh/L=0.600 to Th(Max)    281
Missing Very Strong Refl. ....      0
Beamstop Effected Reflections       0

Space Group Extinctions ......    207


Intensity Distribution [Decay of I/Sigma(I) versus sin(theta)/lambda]
==============================================================================
======
sh  st/l   Ang     #  0.25   1.0   2.0  Percent  Distr. for I .gt. 2.0 *
sig(I)
==============================================================================
======
 1 0.301 1.661   350  99.1  98.3  97.1
***************************************.
 2 0.379 1.318   357  96.9  92.2  87.7
***********************************.....
 3 0.434 1.152   361  97.0  92.0  87.5
***********************************.....
```

```
 4 0.478 1.046   357  95.2  90.2  82.1
*********************************.......
 5 0.515 0.971   348  93.1  85.6  75.9
******************************..........
 6 0.547 0.914   372  90.9  80.9  71.0
****************************............
 7 0.576 0.868   352  88.1  75.9  61.9
*************************...............
 8 0.602 0.830   357  87.4  73.1  52.4
*********************...................
 9 0.626 0.798   351  84.9  66.7  44.2
******************......................
10 0.649 0.771   233  81.5  63.5  36.9
***************.........................
11 0.670 0.747    27  77.8  51.9  29.6
************............................
                                       I                   I
I
                  Percent Observed:   0                  50
100
```

Maximum Percentage of Reflections with I .gt. 2*s(I) in any Resolution Shell 97

Section 7
==
Analysis of Variance (F(obs) and F(calc) from FCF)
==

:: Hmax =   12, Kmax =   13, Lmax =   19

:: Sigma Includes SHELXL WGHT Par.   0.0548   0.1507

:: K = Mean[Fo^2]/Mean[Fc^2] for Group - (Fo^2, Fc^2 from FCF)

```
Fc/Fc(max)      0.000 0.003 0.006 0.010 0.014 0.018 0.024 0.032 0.045
0.074 1.000
Number in Group    349   346   345   348   345   347   346   347   346
346
           GooF  0.924 0.901 1.077 1.108 1.120 1.097 1.071 1.079 0.967
1.078
              K  5.752 1.375 1.049 0.967 0.980 1.020 1.010 1.009 1.018
1.014

Resolution(A)   0.76  0.81  0.84  0.88  0.92  0.98  1.06  1.16  1.33
1.67  7.16
Number in Group    347   347   346   347   346   347   346   347   346
346
           GooF  0.956 1.053 1.057 0.996 0.994 1.019 1.088 0.874 1.139
1.232
```

```
              K  1.059 1.042 1.028 1.018 1.021 1.014 1.027 1.023 1.029
1.009

Resolution Dependence for Fc/Fc(max) .LT.  0.006
Resolution(A)   0.76  0.81  0.84  0.88  0.92  0.98  1.06  1.16  1.33
1.67  7.16
Number in Group    124   113   107    83    74    51    45    45    40
13
           GooF  0.772 0.978 0.933 0.833 0.880 0.854 1.066 0.736 1.013
1.170
              K  1.982 2.580 1.986 1.647 2.213 1.468 2.126 1.532 1.103
1.651

Abs(H)         0    1    2    3    4    5    6    7    8    9   10   11
12
Number       200  404  401  396  377  353  323  280  242  200  155   98
36
PerObs Fo2    71   70   70   64   67   63   62   60   53   49   33   29
14
PerObs Fc2    70   71   71   65   69   65   63   60   56   49   36   34
17

Abs(K)         0    1    2    3    4    5    6    7    8    9   10   11
12   13
Number       183  377  379  368  349  329  302  280  255  215  176  134
83   35
PerObs Fo2    71   69   67   66   64   63   64   60   54   55   44   49
39   34
PerObs Fc2    74   68   69   66   66   63   68   61   50   57   48   49
36   31

Abs(L)         0    1    2    3    4    5    6    7    8    9   10   11
12   13
Number       138  256  278  251  265  236  250  218  228  198  208  175
176  143
PerObs Fo2    67   71   68   68   70   69   65   61   67   56   64   55
60   52
PerObs Fc2    67   73   69   69   71   68   66   64   66   57   63   59
61   55



                     I------------I-----------I------------I-----------I-
-----------I-----------I------------I-----------I
          3.898=Max-
x -
                   I Normal Probability Plot      (S.C.Abrahams and
E.T.Keve (1971). Acta Cryst. A27, 157-165.)           I
                   I
I
                   I Sample Size    =      3465    NPP for (Fobs**2 -
Fcalc**2) / Sigma(Fobs**2)                     x    I
                   I CC             =    0.9983
xx x      I
```

```
          2.874    - Est. Intercept =    0.0341    Sigma Includes SHELXL
WGHT Par.   0.0548   0.1507           xxx         -
                   I Est. Slope     =    1.0101
xxx            I
                   I
xxx              I
                   I
xxxx                I
                   I
xxxxx                   I
          1.851    -
xxxx                       -
                   I
xxxxx                          I
                   I
xxx                              I
                   I
xxxx                                I
                   I
xxxx                                   I
          0.827    -
xxxx                                      -
                   I
xxxxx                                         I
                   I
xxxx                                             I
                   I
xxx                                                I
                   I                                                 xxxx
I
         -0.197=Mid-                                              xxxx
-
                   I                                           xxxx
I
                   I                                        xxxx
I
                   I                                     xxxx
I
                   I                                   xxx
I
         -1.221    -                                xxxx
-
                   I                              xxx
I
                   I                           xxxx
I
                   I                         xxx
I
                   I                      xxxx
I
         -2.245    -                   xxxx
-
                   I                  xx
I
```

```
                   I                xxx
I
                   I              xxx
I
                   I            xxx
I
         -3.269    -           x
-
                   I          x
I
                   I      x xx
I
                   I    x
I
                   I
I
         -4.293=Min- x                                        Vertical:
Experimental Data, Horizontal: Theoretical Data   -
                     I------------I-----------I------------I-----------I-
-----------I-----------I------------I-----------I
                   -3.540                   -1.770
0.000                 1.770                    3.540
```

Section 8
==
======
Check for Unaccounted Twinning with the TwinRotMat Algorithm - N(selec) = 35
==
======
Note: This Analysis is Based on Fc calculated from Coordinates in the CIF.
==
======

No Applicable Twin Law(s) Detected from Fo/Fc Analysis-or Already Accounted for

Section 8
==
======
Check for Unaccounted Twinning with the TwinRotMat Algorithm - N(selec) = 35
==
======
Note: This Analysis is Based on Fc Taken from Fo/Fc File
==
======

No Applicable Twin Law(s) Detected from Fo/Fc Analysis-or Already Accounted for

```
Section 10:
=========================================================================
=======
Analysis of Difference Map Grid Point Density - (MIN = -0.17, MAX =
0.17 eA-3)
=========================================================================
=======
Frequency Plot Sqrt(Frequency) - Average =  0.000, Sigma =  0.037 eA-3
=========================================================================
======
                               Note: F(obs) from FCF and F(calc) from CIF
model
 -0.25         0
 -0.20         0
 -0.15        76 ***
 -0.10      1324 ************
 -0.05     14472 ***************************************
  0.00     33510
************************************************************
  0.05     14628 ****************************************
  0.10      1454 ************
  0.15        72 ***
  0.20         0
  0.25         0

=========================================================================
======
Unique Density Maxima in          Difference Map (CutOff level =     0.10
eA-3)
=========================================================================
======
   #   x     y     z  (e/A^3)     Shortest Contacts within 3.2 Ang.
(Excl. H)
=========================================================================
======
   1 0.589 0.122 0.439  0.17      C11  0.50; C12  1.02; C16  1.59; C1
1.79;
   2 0.813 0.179 0.266  0.15      C34  0.36; C35  1.16; C33  1.51; C2
1.66;
   3 0.594 0.063 0.426  0.14      C11  0.34; C16  1.10; C1   1.56; C12
1.63;
   4 0.772 0.900 0.475  0.14      C1   1.42; O1   2.12; C11  2.52; C24
2.88;
   5 0.965 0.250 0.390  0.13 C-C  C31  0.70; C32  0.79; C36  1.81; C33
1.85;
   6 0.975 0.744 0.142  0.13      C32  0.80; C31  0.94; C33  1.79; C36
1.95;
   7 0.994 0.158 0.330  0.12 C-C  C33  0.84; C32  0.85; C31  1.82; C34
1.83;
   8 0.333 0.712 0.348  0.12      O4   1.22; C2   1.78; C35  2.07; C34
2.17;
```

```
   9 0.231 0.847 0.427  0.12      O1   1.03; C1   1.87; C11  2.54; C12
2.57;
  10 0.459 0.147 0.289  0.12 C-C  C14  0.75; C15  0.81; O2   1.85; C13
1.87;
  11 0.093 0.999 0.157  0.12      C36  1.51; O3   2.13; C31  2.14; C23
2.46;
  12 0.795 0.126 0.241  0.12      C34  0.51; C2   1.00; C33  1.66; C35
1.72;
  13 0.607 0.068 0.372  0.12      C16  0.89; C11  0.92; C15  1.81; C12
1.82;
  14 0.929 0.154 0.377  0.11      C32  0.77; C33  0.97; C31  1.75; C34
1.97;
  15 0.993 0.340 0.407  0.11      C31  0.51; O3   0.91; C36  1.69; C32
1.69;
  16 0.564 0.561 0.205  0.11      C15  0.23; C16  1.43; C14  1.56; O2
2.53;
  17 0.401 0.377 0.359  0.11      C13  1.52; O2   2.00; C14  2.00; O4
2.51;
  18 0.917 0.969 0.372  0.11      C33  1.67; C32  2.42; C22  2.50; C34
2.73;
  19 0.207 0.130 0.215  0.11      C21  1.37; C22  1.58; O2   2.05; C26
2.23;
  20 0.953 0.619 0.044  0.11      C32  1.32; C33  2.21; C31  2.41; O1
2.50;
  21 0.647 0.056 0.456  0.11      C11  0.54; C1   0.94; C16  1.67; C12
1.77;
  22 0.717 0.753 0.373  0.10      C21  0.78; C26  1.12; C22  1.64; O2
1.88;
  23 0.127 0.503 0.170  0.10      C33  1.24; C34  2.00; C32  2.38; C2
2.39;
  24 0.131 0.094 0.192  0.10      C22  1.63; C21  1.98; C23  2.11; C36
2.44;
  25 0.592 0.660 0.170  0.10      C14  0.67; C15  1.02; C13  1.67; O2
1.80;

Density Maxima within  1.2 Angstrom from Atoms (CutOff level =     0.10
eA-3)
==========================================================================
======
Atom      # e/A^3   Ang    # e/A^3   Ang    # e/A^3   Ang    # e/A^3
Ang
==========================================================================
======
O1        9  0.12  1.03:
O3       15  0.11  0.91:
C1       21  0.11  0.94:
C2       12  0.12  1.00:
C11       3  0.14  0.34:   1  0.17  0.50:  21  0.11  0.54:  13  0.12
0.92:
C12       1  0.17  1.02:
C14      25  0.10  0.67:  10  0.12  0.75:
C15      16  0.11  0.23:  10  0.12  0.81:  25  0.10  1.02:
C16      13  0.12  0.89:   3  0.14  1.10:
C21      22  0.10  0.78:
```

```
C26       22  0.10  1.12:
C31       15  0.11  0.51:   5  0.13  0.70:   6  0.13  0.94:
C32       14  0.11  0.77:   5  0.13  0.79:   6  0.13  0.80:   7  0.12
0.85:
C33        7  0.12  0.84:  14  0.11  0.97:
C34        2  0.15  0.36:  12  0.12  0.51:
C35        2  0.15  1.16:
H1         4  0.14  0.71:
H13       17  0.11  1.03:
H15       16  0.11  0.73:
H32       20  0.11  0.57:
H33       23  0.10  0.72:  18  0.11  0.97:
H36       11  0.12  0.78:

============================================================================
======
Unique Density Minima in          Difference Map (CutOff level =    -0.10
eA-3)
============================================================================
======
   #   x     y     z  (e/A^3)     Shortest Contacts within 3.2 Ang.
(Excl. H)
============================================================================
======
   1 0.039 0.246 0.170 -0.17      C22  0.94; C23  1.48; C21  2.27; C34
2.72;
   2 0.244 0.914 0.409 -0.15      O1   0.81; C1   1.61; C11  2.60; C35
2.76;
   3 0.190 0.215 0.123 -0.15      C22  0.89; C21  1.02; C23  1.40; C26
1.57;
   4 0.871 0.399 0.047 -0.15      O1   0.79; C1   1.81; C23  2.76; C11
2.84;
   5 0.740 0.989 0.173 -0.14      C2   0.92; O4   1.49; C34  2.28; O2
2.72;
   6 0.649 0.472 0.017 -0.14      C1   0.80; C11  1.43; O1   1.56; C12
2.39;
   7 0.253 0.029 0.483 -0.13      C1   0.65; O1   1.24; C11  2.08; C16
2.90;
   8 0.629 0.626 0.426 -0.13      C26  0.78; C25  1.18; C21  2.16; C24
2.49;
   9 0.215 0.155 0.081 -0.12      C25  1.00; C26  1.22; C24  1.26; C21
1.59;
  10 0.990 0.261 0.103 -0.12      C23  0.99; C22  1.52; C24  2.32; O1
2.64;
  11 0.180 0.999 0.450 -0.12      O1   0.73; C1   1.22; C11  2.64; C32
2.74;
  12 0.648 0.624 0.479 -0.11      C25  0.91; C26  1.43; C24  2.26; C21
2.75;
  13 0.944 0.366 0.284 -0.11      C36  0.78; C31  1.55; C35  1.57; O3
2.43;
  14 0.250 0.283 0.203 -0.11      C21  0.83; O2   1.06; C22  1.35; C26
2.17;
  15 0.250 0.274 0.397 -0.11      C25  2.27; C24  2.48; C14  2.49; O3
2.51;
```

```
  16 0.801 0.221 0.378 -0.11      C31  1.98; C32  1.98; C33  2.00; C36
2.04;
  17 0.753 0.222 0.357 -0.11      C35  1.92; C12  1.95; C34  1.95; C36
2.15;
  18 0.355 0.212 0.189 -0.11      C21  0.84; O2   1.16; C26  1.31; C14
1.84;
  19 0.636 0.664 0.058 -0.11      C13  2.27; C14  2.28; C1   2.36; C12
2.46;
  20 0.816 0.736 0.315 -0.11      C22  0.62; C21  0.91; O2   1.76; C23
1.93;
  21 0.446 0.999 0.386 -0.10      C16  0.94; C15  1.47; C11  1.96; C14
2.50;
  22 0.896 0.318 0.406 -0.10      C31  0.94; C36  1.49; O3   1.73; C32
1.80;
  23 0.283 0.732 0.085 -0.10      C12  1.17; C11  1.86; C13  2.07; C1
2.56;
  24 0.033 0.128 0.018 -0.10      C24  0.90; C23  1.22; O3   1.30; C31
2.19;
  25 0.618 0.059 0.330 -0.10      C16  1.17; C11  1.58; C15  1.75; C12
2.30;

Density Minima within  1.2 Angstrom from Atoms (CutOff level =    -0.10
eA-3)
==========================================================================
======
Atom      # e/A^3   Ang    # e/A^3   Ang    # e/A^3   Ang    # e/A^3
Ang
==========================================================================
======
O1       11 -0.12  0.73:   4 -0.15  0.79:   2 -0.15  0.81:
O2       14 -0.11  1.06:  18 -0.11  1.16:
C1        7 -0.13  0.65:   6 -0.14  0.80:
C2        5 -0.14  0.92:
C12      23 -0.10  1.17:
C16      21 -0.10  0.94:  25 -0.10  1.17:
C21      14 -0.11  0.83:  18 -0.11  0.84:  20 -0.11  0.91:   3 -0.15
1.02:
C22      20 -0.11  0.62:   3 -0.15  0.89:   1 -0.17  0.94:
C23      10 -0.12  0.99:
C24      24 -0.10  0.90:
C25      12 -0.11  0.91:   9 -0.12  1.00:   8 -0.13  1.18:
C26       8 -0.13  0.78:
C31      22 -0.10  0.94:
C36      13 -0.11  0.78:
H1        7 -0.13  0.60:
H2        5 -0.14  0.46:
H12      23 -0.10  1.18:
H22       1 -0.17  0.71:  20 -0.11  1.12:
H23      10 -0.12  0.75:
H25      12 -0.11  0.75:
H26       8 -0.13  0.89:
#=========================================================================
=======
```

```
# R= 0.0474(  2442), wR2= 0.1292(  3465), S = 1.045      (From CIF+FCF
data)
# R= 0.0474(  2442), wR2= 0.1292(  3465), S = 1.045      (From FCF data
only)
# R= 0.0474(  2443), wR2= 0.1292(  3465), S = 1.045, Npar=  218
==========================================================================
======
PLATON/ASYM-(Version   70316)-[Mode=2] FCF-File Validation for:15-010_3e
==========================================================================
======
For Documentation: http://http://www.platonsoft.nl/FCF-VALIDATION.pdf

Section 1
==========================================================================
======
General Data
==========================================================================
======
Crystal Data From: platon.cif
Fo/Fc   Data From: platon.hkl FCF-TYPE=LIST4
Space Group      : P-1
Wavelength (Ang) :   0.71073
Unit Cell (CIF)  :    7.5560  11.7620  13.8277   95.137  103.256  102.373
SHELX WGHT Pars. :    0.0510   0.1684

Section 2
==========================================================================
======
Reflections with abs((I(obs) - I(calc)) / SigW(I) > 3.0      [I(calc)
from FCF]
==========================================================================
======
   Nr   H   K   L  Theta     I(obs)    I(calc) Sigma(I)   Ratio  SigW(I)
RatioW
--------------------------------------------------------------------------
------
    1   5   0   0  14.42      35.04      24.77     2.14    4.80     3.38
3.04
    2   4   1   0  12.06      27.40      38.26     1.94   -5.60     3.57
-3.05
    3   0   3   0   5.38      11.08       2.68     2.61    3.22     2.80
3.01
    4  -3   4   0   9.79      26.50      17.15     1.36    6.88     2.52
3.72
    5  -2   5   0   9.43      17.32      10.97     0.92    6.90     1.87
3.40
    6   9  -9   1  28.22       3.28      10.76     2.14   -3.50     2.48
-3.02
    7  -5  15   1  28.41       0.55       4.72     0.92   -4.53     1.20
-3.48
    8   2  -3   2   7.77      32.90      46.38     1.66   -8.12     3.79
-3.56
    9  -4  -1   2  11.63      24.80      35.68     1.49   -7.30     3.21
-3.39
```

10	3	3	3	13.26	57.63	43.91	2.51	5.47	4.54	3.02
11	-3	4	3	10.33	8.36	3.00	0.77	6.96	1.21	4.44
12	-1	5	3	10.17	103.55	128.42	3.15	-7.90	8.23	-3.02
13	-6	11	3	23.58	0.56	4.92	0.90	-4.84	1.19	-3.65
14	0	-5	4	10.14	23.17	13.01	1.64	6.20	2.48	4.10
15	-9	-4	4	28.44	0.12	6.60	1.72	-3.77	1.94	-3.34
16	6	-3	4	19.30	55.53	33.98	5.81	3.71	6.72	3.21
17	-2	2	4	7.87	67.88	97.22	2.60	-11.28	6.43	-4.56
18	-2	3	4	8.74	2.37	5.53	0.55	-5.75	1.05	-3.00
19	1	-5	5	11.28	42.81	24.56	2.34	7.80	3.62	5.05
20	2	-5	5	12.29	206.47	168.77	6.34	5.95	12.50	3.02
21	-6	-3	5	18.95	-0.46	3.47	0.90	-4.37	1.10	-3.57
22	1	-3	5	9.38	14.30	8.53	1.00	5.77	1.74	3.31
23	2	-3	5	10.80	128.62	97.89	4.38	7.02	8.23	3.73
24	3	6	5	19.04	0.65	4.22	0.92	-3.88	1.17	-3.04
25	1	-5	6	12.32	7.34	13.26	1.01	-5.86	1.80	-3.28
26	-3	2	6	11.25	20.86	32.12	1.44	-7.82	2.99	-3.77
27	1	2	6	11.61	164.03	133.20	4.00	7.71	9.68	3.18
28	2	-5	7	14.50	130.81	104.41	4.24	6.23	8.39	3.15
29	5	0	7	20.33	3.65	12.38	1.40	-6.24	1.95	-4.49
30	2	-6	8	16.52	38.12	24.06	2.29	6.14	3.50	4.02
31	0	0	8	12.33	39.49	23.20	1.58	10.31	3.07	5.30
32	1	-4	9	15.36	36.54	25.91	2.10	5.06	3.41	3.12
33	0	1	10	15.85	118.86	92.52	5.23	5.04	8.43	3.12
34	-3	5	10	18.29	1.65	7.39	1.27	-4.52	1.62	-3.55
35	1	-5	15	24.75	7.91	0.06	2.34	3.35	2.44	3.22
								--------	--	------

Average = 0.55 0.35

Note: SigW(I) is the SHELXL optimized weight

For I(calc) < 2 Sigma(I): = 1.57 and = 1.12

Section 3

==

Missing Reflections (Asym. Refl. Unit) below sin(th)/lambda = 0.5

==

Nr	H	K	L	sin(th)/lambda	Theta	I(calc)	I(calc)/I(max)
1	-1	1	0	0.073	2.98	348.33	0.02392
2	0	1	0	0.044	1.79*	145.70	0.01000
3	-1	3	0	0.134	5.45	2298.27	0.15780
4	0	-1	1	0.054	2.18*	417.01	0.02863
5	-1	0	1	0.071	2.87	0.51	0.00003
6	0	0	1	0.038	1.53*	22.87	0.00157
7	0	1	1	0.062	2.52*	580.50	0.03986
8	0	1	2	0.092	3.77	63.45	0.00436
9	0	1	3	0.127	5.17	2259.13	0.15511

** Note: I(max) is the maximum I(obs) encountered in the fcf-file **

Starred Reflections have a Theta below Theta(Min) = 2.85

From CIF: Theta(Min) = 2.86

Section 4:

==

Resolution & Completeness Statistics (Cumulative and Friedel Pairs Averaged)

==

Theta	sin(th)/Lambda	Complete	Expected	Measured	Missing
20.82	0.500	0.996	2413	2404	9

23.01	0.550	0.997	3217	3208	9
25.24	0.600	0.998	4174	4165	9
---------- ACTA Min. Res. ---					
27.51	0.650	0.998	5316	5307	9
29.53	0.694	0.928	6466	6002	464

Note: The Reported Completeness refers to the Actual H,K,L Index Range

Section 5

===

R-Value Statistics as a Function of Resolution (in Resolution Shell)

===

Theta	sin(Th)/L	#	R1	wR2	S	Rs	av(I/SigW)	av(I)	av(SigW)
12.38	0.302	529	0.030	0.098	1.336	0.024	12.70	580.50	34.34
15.68	0.380	520	0.042	0.113	1.042	0.054	7.92	71.58	6.30
18.02	0.435	535	0.038	0.112	0.940	0.056	6.81	68.97	6.02
19.90	0.479	543	0.050	0.137	1.000	0.073	5.85	46.11	4.82
21.51	0.516	524	0.061	0.167	0.937	0.107	4.28	24.72	3.43
22.94	0.548	524	0.092	0.251	0.986	0.191	2.86	11.14	2.51
24.22	0.577	543	0.109	0.304	0.952	0.269	2.26	7.34	2.23
25.40	0.603	533	0.128	0.347	0.944	0.321	1.88	6.24	2.20
26.49	0.628	541	0.143	0.399	0.903	0.439	1.50	4.29	2.03
27.52	0.650	518	0.160	0.455	0.939	0.480	1.44	4.00	2.05
28.49	0.671	501	0.175	0.513	0.933	0.569	1.28	3.41	2.05
29.41	0.691	183	0.238	0.546	0.928	0.625	1.13	3.10	2.03
29.53	0.694	8	0.100	0.308	0.714	0.371	1.93	6.42	2.56

R(sig) = sum(sig(I)) / sum(I) = 0.0486

From FCF: R1 = 0.0547(3569), wR2 = 0.1540(6002), S = 1.022

From CIF: R1 = 0.0548(3571), wR2 = 0.1540(6002), S = 1.022, Npar = 300

```
No (SHELXL) Optimized Weights:  wR2 = 0.1067         , S =    1.47

Section 6
======================================================================
======
Summary of Reflection Data in FCF - Note: Friedel Pairs Averaged
======================================================================
======
Total # of Reflections in FCF.   6002  (Hmax = 10, Kmax = 16, Lmax = 19)
Obs
Number above Rep. Theta(Max) .      0
Actual   Theta(Max) (Deg.) ... 29.533  (Hmax = 10, Kmax = 16, Lmax = 19)
Exp
Reported Theta(Max) (Deg.) ... 29.533  (Hmax = 10, Kmax = 16, Lmax = 19)
Rep
Actual   Theta(Min) (Deg.) ...  2.855
Reported Theta(Min) (Deg.) ...  2.855

Unique (Expected) ...........   6466
Unique (in FCF) .............   6002
Observed [I .gt. 2 Sig(I)] ...   3576
Less-Thans ..................   2426
Negative Intensities .........    644
Negative Intensities < - 2 SIG      0

Missing (Total) .............    464
Missing Below Th(Min) ........      4
Missing Th(Min) to STh/L=0.600      5
Missing STh/L=0.600 to Th(Max)    455
Missing Very Strong Refl. ....      0
Beamstop Effected Reflections       0

Space Group Extinctions ......      0


Intensity Distribution [Decay of I/Sigma(I) versus sin(theta)/lambda]
======================================================================
======
sh  st/l   Ang     #  0.25   1.0   2.0  Percent  Distr. for I .gt. 2.0 *
sig(I)
======================================================================
======
 1 0.301 1.661   527  99.4  97.9  96.8
***************************************.
 2 0.379 1.318   516  96.1  91.7  87.2
***********************************.....
 3 0.434 1.152   534  91.8  86.5  80.5
********************************........
 4 0.478 1.046   530  90.0  83.8  79.1
********************************........
 5 0.515 0.971   522  87.7  81.2  69.2
****************************............
```

```
 6 0.547 0.914   530  85.5  76.0  60.6
************************.................
 7 0.576 0.868   537  84.7  70.8  52.9
*********************....................
 8 0.602 0.830   522  80.5  63.6  43.1
*****************........................
 9 0.626 0.798   542  76.8  56.3  35.1
**************...........................
10 0.649 0.771   523  79.0  59.8  33.5
*************............................
11 0.670 0.747   495  78.0  58.6  31.3
*************............................
12 0.689 0.725   213  71.8  49.8  24.4
**********...............................
13 0.708 0.706    11  90.9  81.8  36.4
***************..........................
                                       I                   I
I
                  Percent Observed:   0                   50
100
```

Maximum Percentage of Reflections with I .gt. 2*s(I) in any Resolution Shell 97

Section 7
==
Analysis of Variance (F(obs) and F(calc) from FCF)
==

```
:: Hmax =   10, Kmax =   16, Lmax =   19

:: Sigma Includes SHELXL WGHT Par.   0.0510   0.1684

:: K = Mean[Fo^2]/Mean[Fc^2] for Group - (Fo^2, Fc^2 from FCF)

Fc/Fc(max)     0.000 0.003 0.006 0.009 0.013 0.018 0.023 0.033 0.049
0.086 1.000
Number in Group    601   611   590   603   600   598   601   599   600
599
           GooF  0.880 0.897 0.975 1.035 1.130 1.058 1.030 1.124 1.067
0.995
              K  9.573 1.990 1.270 1.052 1.086 1.046 1.034 1.038 1.017
1.000

Resolution(A)   0.72  0.76  0.80  0.83  0.88  0.93  1.00  1.10  1.26
1.58  7.14
Number in Group    601   600   601   600   600   600   600   601   600
599
           GooF  0.970 0.947 0.927 0.976 0.976 0.997 1.003 0.969 1.029
1.360
```

```
              K  1.296 1.195 1.164 1.121 1.031 1.009 1.010 1.017 1.002
0.998

Resolution Dependence for Fc/Fc(max) .LT.  0.006
Resolution(A)    0.72  0.76  0.80  0.83  0.88  0.93  1.00  1.10  1.26
1.58  7.14
Number in Group     217   198   200   170   127    91    71    72    51
15
           GooF  0.899 0.839 0.849 0.918 0.877 0.994 0.823 0.799 0.754
1.197
              K  5.044 3.488 2.901 3.279 1.188 2.368 0.604 1.045 1.576
3.041

Abs(H)          0     1     2     3     4     5     6     7     8     9    10
Number        431   866   849   809   749   671   579   469   341   199    39
PerObs Fo2     58    58    54    57    53    48    42    36    28    16    13
PerObs Fc2     55    58    54    57    54    48    41    33    26    16    13

Abs(K)          0     1     2     3     4     5     6     7     8     9    10    11
12   13
Number        280   556   559   547   529   514   490   449   427   384   344   299
242  186
PerObs Fo2     64    59    61    56    57    54    51    49    52    44    38    28
26   15
PerObs Fc2     63    62    62    55    57    52    51    51    47    42    39    29
31   11

Abs(L)          0     1     2     3     4     5     6     7     8     9    10    11
12   13
Number        236   478   476   468   459   444   431   409   389   371   343   319
286  256
PerObs Fo2     56    60    53    58    54    53    53    52    47    53    49    43
40   30
PerObs Fc2     59    61    55    57    55    53    53    54    47    51    48    42
39   29



                     I------------I-----------I------------I-----------I-
-----------I-----------I------------I-----------I
          5.299=Max-
x -
                   I Normal Probability Plot       (S.C.Abrahams and
E.T.Keve (1971). Acta Cryst. A27, 157-165.)       x    I
                   I
I
                   I Sample Size    =      6002    NPP for (Fobs**2 -
Fcalc**2) / Sigma(Fobs**2)                x      I
                   I CC             =    0.9978
I
          4.067    - Est. Intercept =    0.0612    Sigma Includes SHELXL
WGHT Par.   0.0510   0.1684             xx       -
                   I Est. Slope     =    0.9928
x         I
```

```
                   I
I
                   I
xx          I
                   I
xxx            I
          2.834    -
x               -
                   I
xxx                I
                   I
xxx                  I
                   I
xxxx                    I
                   I
xxxxx                       I
          1.601    -
xxxx                           -
                   I
xxxxx                              I
                   I
xxxxx                                  I
                   I
xxxxx                                      I
                   I
xxxx                                          I
          0.369=Mid-
xxxxx                                             -
                   I
xxxx                                                 I
                   I                                             xxxxx
I
                   I                                          xxxx
I
                   I                                       xxxx
I
         -0.864    -                                    xxxx
-
                   I                                xxxxx
I
                   I                             xxxx
I
                   I                          xxxx
I
                   I                       xxxx
I
         -2.096    -                    xxxx
-
                   I                  xxx
I
                   I               xxx
I
                   I            xxx
I
```

```
                    I            xx
I
         -3.329    -           x
-
                    I       xxxx
I
                    I      x
I
                    I
I
                    I
I
         -4.562=Min- x  x                                     Vertical:
Experimental Data, Horizontal: Theoretical Data   -
                      I------------I-----------I------------I-----------I-
-----------I-----------I------------I-----------I
                    -3.683                   -1.841
0.000                     1.841                    3.683
```

Section 8
==
======
Check for Unaccounted Twinning with the TwinRotMat Algorithm - N(selec) = 48
==
======
Note: This Analysis is Based on Fc calculated from Coordinates in the CIF.
==
======

No Applicable Twin Law(s) Detected from Fo/Fc Analysis-or Already Accounted for

Section 8
==
======
Check for Unaccounted Twinning with the TwinRotMat Algorithm - N(selec) = 47
==
======
Note: This Analysis is Based on Fc Taken from Fo/Fc File
==
======

No Applicable Twin Law(s) Detected from Fo/Fc Analysis-or Already Accounted for

Section 10:

```
==========================================================================
=======
Analysis of Difference Map Grid Point Density - (MIN = -0.16, MAX =
0.23 eA-3)
==========================================================================
=======
Frequency Plot Sqrt(Frequency) - Average =  0.000, Sigma =  0.031 eA-3
==========================================================================
======
                              Note: F(obs) from FCF and F(calc) from CIF
model
 -0.25         0
 -0.20         0
 -0.15        22 *
 -0.10      1352 ********
 -0.05     25029 **********************************
  0.00     78620
************************************************************
  0.05     24572 **********************************
  0.10      1333 ********
  0.15       108 **
  0.20        34 *
  0.25         2
  0.30         0

==========================================================================
======
Unique Density Maxima in          Difference Map (CutOff level =     0.10
eA-3)
==========================================================================
======
   #   x     y     z  (e/A^3)     Shortest Contacts within 3.2 Ang.
(Excl. H)
==========================================================================
======
   1 0.894 0.557 0.439  0.23      C3   1.47; O4   2.17; C44  2.51; C43
2.83;
   2 0.597 0.538 0.205  0.15      O1   1.16; C1   1.53; C11  2.23; C16
2.58;
   3 0.371 0.887 0.109  0.14      C22  0.80; C21  0.97; C23  1.80; C26
1.93;
   4 0.962 1.008 0.251  0.14 C-C  C2   0.67; C31  0.87; C5   1.82; C4
1.87;
   5 0.058 0.998 0.327  0.13 C-C  C31  0.52; C36  0.88; C32  1.74; C2
1.80;
   6 0.148 0.999 0.332  0.13      C36  0.87; C31  1.04; C35  1.80; C32
1.93;
   7 0.249 0.902 0.421  0.12      C34  0.85; C35  1.08; C33  1.71; C36
1.97;
   8 0.993 0.092 0.097  0.12      C5   0.95; C2   1.97; C4   2.50; C24
2.66;
   9 0.243 0.120 0.362  0.12      C36  1.33; C31  2.19; C35  2.29; C46
2.66;
```

```
  10 0.205 0.823 0.078  0.11      O2   1.14; C21  1.28; C22  1.63; C14
1.73;
  11 0.598 0.049 0.262  0.11      C23  1.44; C24  2.22; C4   2.39; C22
2.42;
  12 0.817 1.001 0.187  0.11 C-C  C2   0.75; C24  0.79; C4   1.83; C23
1.88;
  13 0.156 0.122 0.206  0.11      C5   1.19; C2   1.92; C4   2.25; C31
2.53;
  14 0.522 0.421 0.177  0.11      O1   0.28; C1   1.40; C11  2.64; C16
3.18;
  15 0.969 0.794 0.206  0.10      C32  1.24; C33  2.11; C31  2.25; C15
2.65;
  16 0.402 0.393 0.458  0.10      C44  0.69; C45  0.99; C43  1.66; C3
1.93;
  17 0.629 0.406 0.154  0.10      C1   0.78; O1   0.86; C11  1.97; C16
2.74;
  18 0.635 0.986 0.174  0.10 C-C  C24  0.87; C23  0.90; C25  1.89; C22
1.95;

Density Maxima within  1.2 Angstrom from Atoms (CutOff level =     0.10
eA-3)
==========================================================================
======
Atom      # e/A^3   Ang    # e/A^3   Ang    # e/A^3   Ang    # e/A^3
Ang
==========================================================================
======
O1       14  0.11  0.28:  17  0.10  0.86:   2  0.15  1.16:
O2       19  0.10  0.12:  10  0.11  1.14:
C1       17  0.10  0.78:
C2        4  0.14  0.67:  12  0.11  0.75:
C5        8  0.12  0.95:  13  0.11  1.19:
C21       3  0.14  0.97:
C22       3  0.14  0.80:
C23      18  0.10  0.90:
C24      12  0.11  0.79:  18  0.10  0.87:
C31       5  0.13  0.52:   4  0.14  0.87:   6  0.13  1.04:
C34       7  0.12  0.85:
C35       7  0.12  1.08:
C36       6  0.13  0.87:   5  0.13  0.88:
C44      16  0.10  0.69:
C45      16  0.10  0.99:
H3        1  0.23  0.54:
H5A       8  0.12  0.14:
H5C      13  0.11  0.52:
H23      11  0.11  1.01:
H32      15  0.10  0.75:
H36       9  0.12  0.90:

==========================================================================
======
Unique Density Minima in          Difference Map (CutOff level =    -0.10
eA-3)
```

```
================================================================================
======
   #   x     y     z  (e/A^3)     Shortest Contacts within 3.2 Ang.
(Excl. H)
================================================================================
======
   1 0.222 0.768 0.043 -0.16      O2   0.46; C21  1.07; C14  1.40; C22
1.99;
   2 0.653 0.216 0.440 -0.14      O3   0.81; C41  0.85; C46  1.56; C34
2.13;
   3 1.007 0.764 0.297 -0.14      C33  0.89; C32  1.08; C34  2.22; C31
2.41;
   4 0.882 0.197 0.279 -0.13      C4   0.66; C2   2.17; C24  3.00; C31
3.04;
   5 0.507 0.360 0.128 -0.13      O1   1.01; C1   1.69; C11  2.81; C26
2.88;
   6 0.100 0.089 0.364 -0.12      C36  0.83; C31  1.54; C35  2.16; C2
2.18;
   7 0.433 0.126 0.017 -0.12      C26  0.80; C25  1.14; C21  2.13; C24
2.45;
   8 0.132 0.426 0.377 -0.12      O4   0.68; C3   1.23; C44  1.97; C45
2.29;
   9 0.099 0.451 0.472 -0.12      C3   0.65; O4   1.06; C44  2.11; C43
3.01;
  10 0.403 0.354 0.346 -0.12      C45  0.61; C46  1.37; C44  1.97; C41
2.63;
  11 0.184 0.080 0.437 -0.12      C36  0.84; C35  1.37; C31  2.20; C34
2.66;
  12 0.735 0.667 0.078 -0.12      C16  0.95; C15  1.48; C11  2.10; C14
2.65;
  13 0.108 0.343 0.456 -0.11      C3   0.98; C44  1.53; O4   1.74; C43
2.34;
  14 0.595 0.436 0.488 -0.11      C45  1.79; C46  1.88; C44  2.00; C3
2.06;
  15 0.863 0.198 0.001 -0.11      O2   0.75; C21  1.71; C14  1.74; C15
2.31;
  16 0.698 0.293 0.471 -0.11      C41  0.77; O3   1.23; C46  1.46; C42
1.94;
  17 0.230 0.456 0.487 -0.11      C3   0.50; C44  1.38; O4   1.47; C43
2.33;
  18 0.432 0.874 0.218 -0.11      C22  0.74; C23  1.24; C21  2.09; C24
2.55;
  19 0.934 0.192 0.216 -0.11      C4   0.76; C2   1.88; C5   2.33; C24
2.84;
  20 0.490 0.231 0.001 -0.10      C26  0.85; C21  1.72; C25  1.81; O2
2.46;
  21 0.281 0.801 0.482 -0.10      O3   0.71; C34  1.16; C41  1.24; C42
1.92;
  22 0.205 0.329 0.003 -0.10      C15  0.73; C16  1.40; C14  2.08; C11
2.69;
  23 0.923 0.939 0.229 -0.10      C31  1.00; C2   1.12; C32  1.47; C24
1.67;
  24 0.469 0.484 0.191 -0.10      O1   0.94; C1   1.99; C11  2.98; C13
3.12;
```

```
Density Minima within  1.2 Angstrom from Atoms (CutOff level =    -0.10
eA-3)
======================================================================
======
Atom      # e/A^3   Ang    # e/A^3   Ang    # e/A^3   Ang    # e/A^3
Ang
======================================================================
======
O1       24 -0.10  0.94:   5 -0.13  1.01:
O2        1 -0.16  0.46:  15 -0.11  0.75:
O3       21 -0.10  0.71:   2 -0.14  0.81:
O4        8 -0.12  0.68:   9 -0.12  1.06:
C2       23 -0.10  1.12:
C3       17 -0.11  0.50:   9 -0.12  0.65:  13 -0.11  0.98:
C4        4 -0.13  0.66:  25 -0.10  0.71:  19 -0.11  0.76:
C15      22 -0.10  0.73:
C16      12 -0.12  0.95:
C21       1 -0.16  1.07:
C22      18 -0.11  0.74:
C25       7 -0.12  1.14:
C26       7 -0.12  0.80:  20 -0.10  0.85:
C31      23 -0.10  1.00:
C32       3 -0.14  1.08:
C33       3 -0.14  0.89:
C34      21 -0.10  1.16:
C36       6 -0.12  0.83:  11 -0.12  0.84:
C41      16 -0.11  0.77:   2 -0.14  0.85:
C45      10 -0.12  0.61:
H3        9 -0.12  0.79:  17 -0.11  1.04:
H4A       4 -0.13  0.78:
H4B      19 -0.11  0.70:   4 -0.13  1.08:
H4C      25 -0.10  0.68:  19 -0.11  0.98:   4 -0.13  1.04:
H15      22 -0.10  0.64:
H16      12 -0.12  1.02:
H22      18 -0.11  0.85:
H26      20 -0.10  0.95:   7 -0.12  0.98:
H33       3 -0.14  1.07:
H36       6 -0.12  0.53:  11 -0.12  0.73:
H45      10 -0.12  0.64:
#=====================================================================
=======
# R= 0.0547(  3569), wR2= 0.1540(  6002), S = 1.022      (From CIF+FCF
data)
# R= 0.0547(  3569), wR2= 0.1540(  6002), S = 1.022      (From FCF data
only)
# R= 0.0548(  3571), wR2= 0.1540(  6002), S = 1.022, Npar=  300
```

checkCIF/PLATON report

Structure factors have been supplied for datablock(s) 15-008_3a, 15-010_3e

THIS REPORT IS FOR GUIDANCE ONLY. IF USED AS PART OF A REVIEW PROCEDURE FOR PUBLICATION, IT SHOULD NOT REPLACE THE EXPERTISE OF AN EXPERIENCED CRYSTALLOGRAPHIC REFEREE.

No syntax errors found. CIF dictionary Interpreting this report

Datablock: 15-008_3a

```
Bond precision:  C-C = 0.0021 A          Wavelength=0.71073

Cell:            a=9.7949(4)     b=10.6069(5)       c=15.1758(6)
                 alpha=90        beta=97.783(4)     gamma=90
Temperature:     294 K

                Calculated                Reported
Volume          1562.14(12)               1562.14(12)
Space group     P 21/c                    P 21/c
Hall group      -P 2ybc                   -P 2ybc
Moiety formula  C20 H14 O4                C20 H14 O4
Sum formula     C20 H14 O4                C20 H14 O4
Mr              318.31                    318.31
Dx,g cm-3       1.354                     1.353
Z               4                         4
Mu (mm-1)       0.094                     0.094
F000            664.0                     664.0
F000'           664.36
h,k,lmax        12,13,20                  12,13,19
Nref            3748                      3465
Tmin,Tmax       0.957,0.987               0.964,0.990
Tmin'           0.957

Correction method= # Reported T Limits: Tmin=0.964 Tmax=0.990
AbsCorr = ANALYTICAL

Data completeness= 0.924          Theta(max)= 27.962

R(reflections)= 0.0474( 2443)     wR2(reflections)= 0.1292( 3465)

S = 1.045                   Npar= 218
```

The following ALERTS were generated. Each ALERT has the format
test-name_ALERT_alert-type_alert-level.
Click on the hyperlinks for more details of the test.

Alert level C

```
PLAT906_ALERT_3_C Large K value in the Analysis of Variance ......      5.752 Check
```

Alert level G

```
PLAT066_ALERT_1_G Predicted and Reported Tmin&Tmax Range Identical          ? Check
PLAT910_ALERT_3_G Missing # of FCF Reflection(s) Below Theta(Min)           3 Note
PLAT912_ALERT_4_G Missing # of FCF Reflections Above STh/L=  0.600        281 Note
PLAT978_ALERT_2_G Number C-C Bonds with Positive Residual Density           3 Note
```

```
   0 ALERT level A = Most likely a serious problem - resolve or explain
   0 ALERT level B = A potentially serious problem, consider carefully
   1 ALERT level C = Check. Ensure it is not caused by an omission or oversight
   4 ALERT level G = General information/check it is not something unexpected

   1 ALERT type 1 CIF construction/syntax error, inconsistent or missing data
   1 ALERT type 2 Indicator that the structure model may be wrong or deficient
   2 ALERT type 3 Indicator that the structure quality may be low
   1 ALERT type 4 Improvement, methodology, query or suggestion
   0 ALERT type 5 Informative message, check
```

Datablock: 15-010_3e

```
Bond precision:  C-C = 0.0027 A         Wavelength=0.71073

Cell:          a=7.5560(9)        b=11.762(2)         c=13.8277(18)
               alpha=95.137(12)   beta=103.256(10)    gamma=102.373(12)
Temperature:   294 K
```

	Calculated	Reported
Volume	1155.9(3)	1155.9(3)
Space group	P -1	P -1
Hall group	-P 1	-P 1
Moiety formula	C29 H24 O4	C29 H24 O4
Sum formula	C29 H24 O4	C29 H24 O4
Mr	436.48	436.48
Dx,g cm-3	1.254	1.254
Z	2	2
Mu (mm-1)	0.083	0.083
F000	460.0	460.0
F000’	460.22	
h,k,lmax	10,16,19	10,16,19
Nref	6466	6002
Tmin,Tmax	0.947,0.967	0.915,1.000
Tmin’	0.947	

Correction method= # Reported T Limits: Tmin=0.915 Tmax=1.000
AbsCorr = MULTI-SCAN

```
Data completeness= 0.928          Theta(max)= 29.533

R(reflections)= 0.0548( 3571)     wR2(reflections)= 0.1540( 6002)

S = 1.022                  Npar= 300
```

The following ALERTS were generated. Each ALERT has the format
test-name_ALERT_alert-type_alert-level.
Click on the hyperlinks for more details of the test.

Alert level C

```
PLAT241_ALERT_2_C High   'MainMol' Ueq as Compared to Neighbors of         O2 Check
PLAT241_ALERT_2_C High   'MainMol' Ueq as Compared to Neighbors of         O3 Check
PLAT906_ALERT_3_C Large K value in the Analysis of Variance ......      9.573 Check
PLAT911_ALERT_3_C Missing # FCF Refl Between THmin & STh/L=  0.600          5 Report
```

Alert level G

```
PLAT063_ALERT_4_G Crystal Size Likely too Large for Beam Size ....       0.65 mm
PLAT910_ALERT_3_G Missing # of FCF Reflection(s) Below Theta(Min)           4 Note
PLAT912_ALERT_4_G Missing # of FCF Reflections Above STh/L=  0.600        455 Note
PLAT978_ALERT_2_G Number C-C Bonds with Positive Residual Density           4 Note
```

0 **ALERT level A** = Most likely a serious problem - resolve or explain
0 **ALERT level B** = A potentially serious problem, consider carefully
4 **ALERT level C** = Check. Ensure it is not caused by an omission or oversight
4 **ALERT level G** = General information/check it is not something unexpected

0 ALERT type 1 CIF construction/syntax error, inconsistent or missing data
3 ALERT type 2 Indicator that the structure model may be wrong or deficient
3 ALERT type 3 Indicator that the structure quality may be low
2 ALERT type 4 Improvement, methodology, query or suggestion
0 ALERT type 5 Informative message, check

It is advisable to attempt to resolve as many as possible of the alerts in all categories. Often the minor alerts point to easily fixed oversights, errors and omissions in your CIF or refinement strategy, so attention to these fine details can be worthwhile. In order to resolve some of the more serious problems it may be necessary to carry out additional measurements or structure refinements. However, the purpose of your study may justify the reported deviations and the more serious of these should normally be commented upon in the discussion or experimental section of a paper or in the "special_details" fields of the CIF. checkCIF was carefully designed to identify outliers and unusual parameters, but every test has its limitations and alerts that are not important in a particular case may appear. Conversely, the absence of alerts does not guarantee there are no aspects of the results needing attention. It is up to the individual to critically assess their own results and, if necessary, seek expert advice.

Publication of your CIF in IUCr journals

A basic structural check has been run on your CIF. These basic checks will be run on all CIFs submitted for publication in IUCr journals (*Acta Crystallographica*, *Journal of Applied Crystallography*, *Journal of Synchrotron Radiation*); however, if you intend to submit to *Acta Crystallographica Section C* or *E* or *IUCrData*, you should make sure that full publication checks are run on the final version of your CIF prior to submission.

Publication of your CIF in other journals

Please refer to the *Notes for Authors* of the relevant journal for any special instructions relating to CIF submission.

PLATON version of 07/03/2016; check.def file version of 02/03/2016

Datablock 15-008_3a - ellipsoid plot

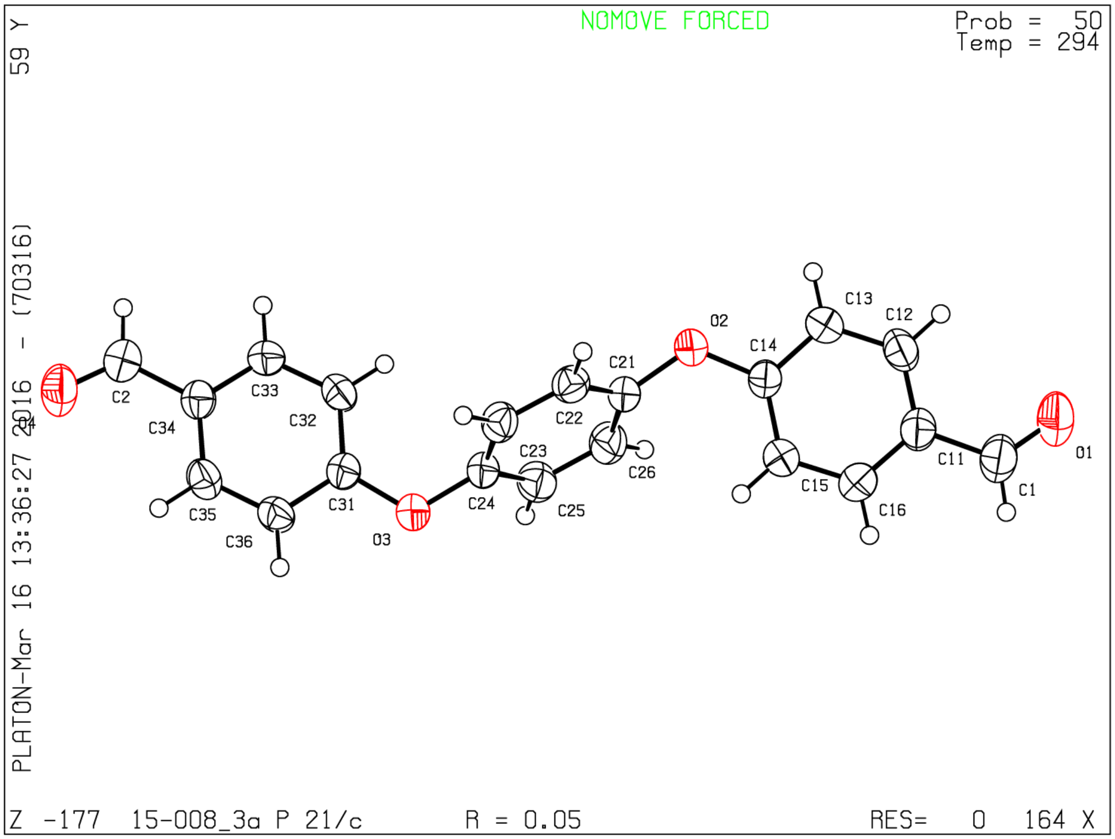

Datablock 15-010_3e - ellipsoid plot

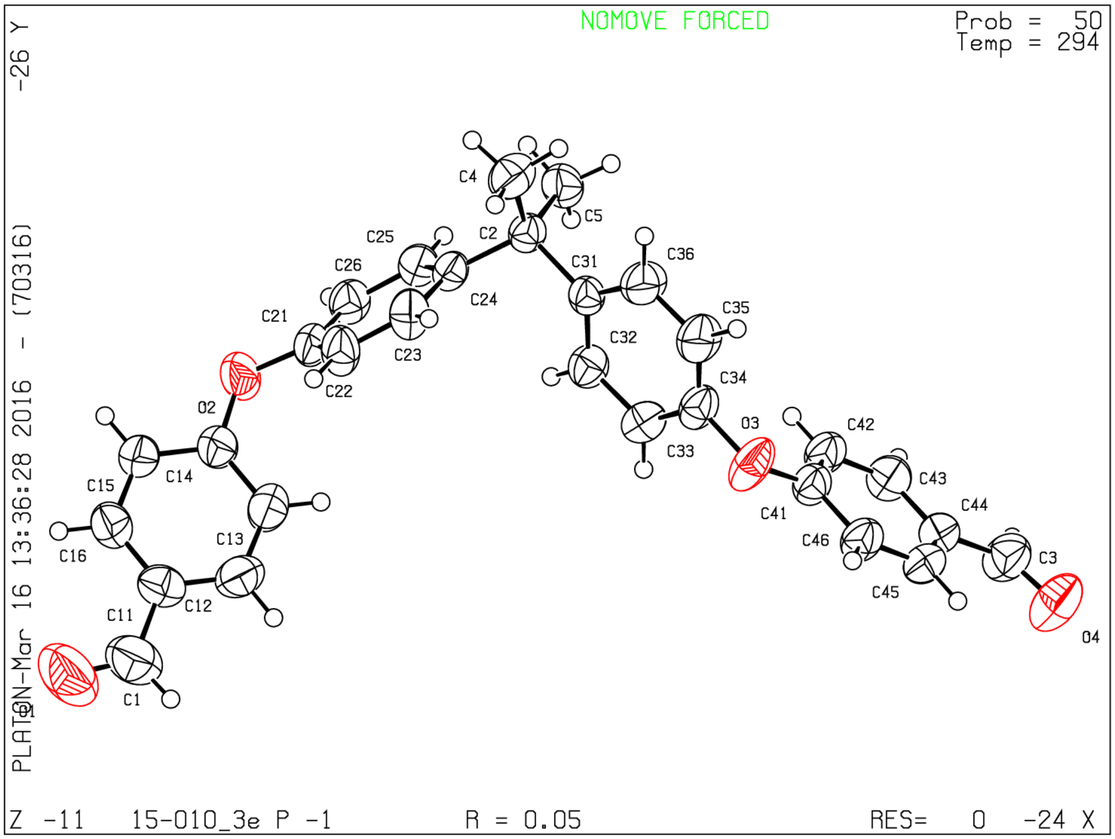

Supplement: TDMP_1231042_Supplementary_Material.pdf [file TDMP_A_1231042_SM3378.pdf]
